# Exploring meaningful outcome domains of recovery following lower limb amputation (LLA) and prosthetic rehabilitation in low- and middle-income (LMIC) settings: a qualitative systematic review

**DOI:** 10.1136/bmjopen-2025-109817

**Published:** 2026-01-28

**Authors:** Caroline Jean Fewins, Chantel Ostler, Margaret Donovan-Hall

**Affiliations:** 1 Hampshire and Isle of Wight Healthcare NHS Foundation Trust, Southampton, England, UK; 2 School of Health Sciences, Faculty of Environmental and Life Sciences, University of Southampton, Southampton, UK; 3 Portsmouth Enablement Centre, Portsmouth Hospitals University NHS Trust, Portsmouth, England, UK

**Keywords:** Systematic Review, QUALITATIVE RESEARCH, Amputation, Surgical, REHABILITATION MEDICINE, Lower Extremity

## Abstract

**Abstract:**

**Objectives:**

To identify outcome domains of importance to adults undergoing prosthetic rehabilitation following lower limb amputation in low- and middle-income countries (LMICs), based on their lived experiences described in qualitative literature.

**Design:**

Systematic review and qualitative synthesis informed by a critical realist perspective and reported according to ENTREQ (Enhancing Transparency in Reporting the Synthesis of Qualitative Research) guidelines.

**Data sources:**

CINAHL, PsycInfo, Web of Science and Trip databases were searched from inception to April 2024.

**Eligibility criteria:**

We included qualitative studies exploring the views and experiences of adults (≥18 years) using lower limb prosthesis in LMICs (World Bank definition). Studies including upper limb amputees, non-prosthetic users, mixed samples that could not be disaggregated or not reporting first-person accounts were excluded.

**Data extraction and synthesis:**

Two reviewers independently screened studies using predefined criteria. Data were extracted from results sections, including participant quotations and author interpretations. Reflexive thematic analysis was conducted to identify outcome domains across studies. Study quality was appraised using the CASP (Critical Appraisal Skills Programme) qualitative checklist; no studies were excluded based on quality.

**Results:**

Five studies involving 55 participants from Nepal, Kenya, Cambodia, Bangladesh and Kiribati met the inclusion criteria. Four outcome domains were identified: (1) The importance of a prosthesis: highlighting access, socket comfort, durability and functional suitability; (2) valued activities: particularly the importance of work and participation in daily living tasks; (3) acceptance following limb loss: encompassing community participation and self and social acceptance; and (4) independence: including reduced reliance on family and greater control over daily life. Across settings, participants emphasised prosthesis durability, work participation and culturally relevant function.

**Conclusion:**

Evidence on meaningful outcomes of prosthetic rehabilitation in LMICs is extremely limited. Findings indicate that access to a comfortable and durable prosthesis enabling work and daily living is central to recovery, alongside social acceptance and independence. These domains may provide initial insights into outcome measurement and development in low-resource settings. Further primary research across diverse LMIC contexts is urgently needed.

**Strengths and limitations of this study**

- A comprehensive search strategy was applied across multiple databases, chosen for their complete indexing of qualitative methodologies, as well as grey literature sources, supplemented by citation-chaining and author contact.
- A researcher positionality statement is included alongside information about the background of the research team and reflexive practices.
- Studies were only included if they provided first-person qualitative data to minimise the impact of multiple researcher interpretations on the findings.
- No review protocol was registered, and 80% of screening and selection and all data extraction were completed by a single reviewer.
- None of the included studies focused on outcome domains of importance, requiring the research team to interpret findings in the context of a different research question.

## Background

 Understanding what defines successful rehabilitation following lower limb amputation (LLA) is key to supporting functional recovery, independence and quality of life. Outcomes are shaped by pre-amputation health, mobility, prosthesis use and psychosocial well-being.^1^ Establishing clear rehabilitation goals enables healthcare providers to tailor therapy and support services, while incorporating patient perspectives ensures care is relevant and person-centred. Evaluating outcomes like mobility, pain management and community participation from the patient’s viewpoint enables a more accurate and holistic assessment of rehabilitation effectiveness.^2^

Although individual rehabilitation centres may use their own service-level indicators to monitor rehabilitation outcomes, the studies in this field use varied and often implicit definitions of what constitutes successful prosthesis use from the patient perspective. Emerging studies have begun identifying outcomes that matter most to individuals with LLA. McDonald *et al*^3^ and Schaffalitzky *et al*^4 5^ found that patients valued balance, independence and adjustment—often differing from clinicians’ priorities. Similarly, Ostler *et al*^6^ and McDonald *et al*^3^ developed conceptual models in England and the USA to guide outcome measurement and rehabilitation focus.

The International Classification of Functioning, Disability and Health (ICF), developed by the WHO, is used to define outcome domains in prosthetic rehabilitation by classifying health across impairments, activity limitations and contextual factors.^7^ Xu developed an ICF core set with input from clinicians and patients in Australia and China, while Clarke *et al*^8^ used the ICF in a Delphi study to identify key outcomes. However, Xu^9^ noted that amputation-specific concerns such as socket comfort and acceptance were missing, potentially limiting the inclusion of patient perspectives.

Despite growing research on prosthetic rehabilitation outcome domains, no studies have focused exclusively on low- and middle-income countries (LMICs), where an estimated 30–40 million people live with limb loss.^10^ Most evidence comes from high-income countries (HICs), despite the greater need for insight in LMICs where access to assistive technology is limited. A rapid review on type 2 diabetes showed that outcome domains of importance to patients differ between settings, with LMICs emphasising life impact factors such as role functioning (eg, ability to work, fulfil household responsibilities or maintain social roles).^11^

Ostler *et al*^2^ conducted a systematic review of 40 studies from 15 countries, including some LMICs, to develop the ECLIPSE (the ECLIPSE model of mEaningful outCome domains of Lower lImb ProSthetic rEhabilitation) model for patient-centred prosthetic rehabilitation outcomes. Drawing on 539 user experiences, the model aimed for global relevance, yet only three studies (5.7% of participants) were from LMICs, limiting its transferability and underscoring the continued lack of insight into successful rehabilitation in low-resource settings. Furthermore, combining these views within a global model may drown out the lived experience of those in LMICs and disregard social and cultural factors which shape patient priorities for recovery. Identifying outcome domains of importance to patients living in LMICs could better guide resource allocation and enhance value for low-resource prosthetic services, as patient-centred outcome frameworks have been shown to improve relevance and prioritisation in prosthetic and assistive technology programmes.^10^,^13^ Therefore, this study aimed to conduct a systematic review and qualitative synthesis of research exploring the experiences of individuals in LMICs with LLA and prosthetic rehabilitation, to identify outcome domains of importance specific to these settings.

## Methods

### Research design

A systematic review and qualitative synthesis were conducted, following Preferred Reporting Items for Systematic Reviews and Meta-Analyses guidelines,^14^ to identify outcome domains important to prosthesis users in LMICs. Given the limited number of relevant studies identified in prior work,^2^ the search included all available literature from inception. Qualitative studies were prioritised for their depth in capturing lived experiences and patient-centred insights. The review was reported according to Enhancing Transparency in Reporting the Synthesis of Qualitative Research (ENTREQ) guidelines.^15^

This review was undertaken from a critical realist perspective which assumes that participants’ accounts reflect real experiences while also being shaped by wider social, cultural and structural influences. This perspective informed the analysis by guiding us to interpret both the explicit descriptions within each study and the underlying contextual factors that may shape outcome priorities across LMIC settings.^16^

### Search strategy

A systematic search strategy was used to identify all relevant studies and ensure comprehensive data inclusion. Search terms were defined using the SPIDER framework, an adaptation of PICO (Population, Intervention, Comparator, Outcome) for qualitative reviews (table 1).

**Table 1:** SPIDER framework to define the review search terms

**S** Sample	Adults with LLA
**PI** Phenomenon of interest	Use of a prosthesis following LLA
**D** Design	Any qualitative approach
**E** Evaluation	Views and experiences
**R** Research type	Qualitative

LLA, lower limb amputation.

To capture all relevant qualitative studies involving people with LLA, LMIC status was not included in search terms due to inconsistent indexing. Instead, the country setting was assessed during screening, with LMIC classification applied as an inclusion/exclusion criterion.

With support from a knowledge specialist, searches were conducted in CINAHL, PsycInfo and Web of Science—databases recommended for qualitative syntheses.^17 18^ Grey literature was searched via the Trip database. Databases were searched from April 2024 back to inception, expanding on the scope of the authors’ previous review.^2^ The strategy is summarised in table 2.

**Table 2:** Search strategy

Database	Syntax
CINAHL	((Amput* OR prosthe* OR “limb loss” OR “artificial limb*”) OR (MH "Amputation" OR MH "Above-Knee Amputation" OR MH "Amputation Stumps" OR MH "Below-Knee Amputation" OR MH "Disarticulation" OR MH "Hemipelvectomy") OR (MH "Prosthesis Design" OR MH "Limb Prosthesis")) **AND** ((“lower limb*” OR leg*) OR (MH "Lower Extremity" OR MH "Ankle" OR MH "Hip" OR MH "Knee" OR MH "Leg" OR MH "Thigh") OR (MH "Leg")) **AND** ((Qualitative OR experience* OR interview* OR “grounded theor*” OR phenomenolog* OR “focus group*” OR narrative OR “thematic analysis” OR “Action research” OR ethnograph*) OR (MH "Qualitative Studies" OR MH "Action Research" OR MH "Ethnographic Research" OR MH "Ethnological Research" OR MH "Ethnonursing Research" OR MH "Grounded Theory" OR MH "Naturalistic Inquiry" OR MH "Phenomenological Research") OR (MH "Life Experiences" OR MH "Work Experiences") OR (MH "Semi-Structured Interview" OR MH "Interview Guides" OR MH "Unstructured Interview" OR MH "Unstructured Interview Guides" OR MH "Structured Interview" OR MH "Structured Interview Guides" OR MH "Interviews") OR (MH “Focus groups”) OR (MH “Narrative medicine”) OR (MH “Thematic analysis”))
PsycInfo	((Amput* OR prosthe* OR “limb loss” OR “artificial limb*”) OR (DE "Amputation" OR DE "Prostheses" OR DE "Phantom Limbs")) **AND** ((“lower limb*” OR leg*) OR DE "Thigh" OR DE "Ankle" OR DE "Knee")) **AND** ((Qualitative OR experience* OR interview* OR “grounded theor*” OR phenomenolog* OR “focus group*” OR narrative OR “thematic analysis” OR “Action research” OR ethnograph*) OR (DE "Focus Group Interview" OR DE "Focus Group" OR DE "Grounded Theory" OR DE "Interpretative Phenomenological Analysis" OR DE "Narrative Analysis" OR DE "Semi-Structured Interview" OR DE "Thematic Analysis" OR DE "Phenomenology") OR (DE "Experiences (Events)" OR DE "Life Changes") OR (DE "Action Research") OR (DE "Ethnography"))
Web of Science	(Amput* OR prosthe* OR “limb loss” OR “artificial limb*”) **AND** (“lower limb*” OR leg*) **AND** (Qualitative OR experience* OR interview* OR “grounded theor*” OR phenomenolog* OR “focus group*” OR narrative OR “thematic analysis” OR “Action research” OR ethnograph*)
Tric database (Grey literature)	Amputation **AND** Prosthesis **AND** qualitative

### Screening process

Study selection was conducted using Rayyan.^19^ After removing duplicates, titles/abstracts were screened using the inclusion and exclusion criteria set out in box 1. Two reviewers (CJF and CO) independently screened 20% of articles to establish consensus, then each screened half of the remaining articles. Disagreements were discussed, with four papers requiring input from a third reviewer (MD-H). The same approach was applied for full-text screening, with undecided cases resolved through group discussion. Mixed-methods studies were included if first-person qualitative data could be clearly identified and extracted. Grey literature sources were searched using the Trip database applying the same screening and selection approach, and inclusion and exclusion criteria. The reference lists of all full-text studies were screened for additional papers. Researchers active in prosthetic rehabilitation in LMICs were also contacted to identify unpublished work.

Box 1 **Inclusion and exclusion criteria**

**Inclusion criteria**

- Adult populations 18 years and older.
- Included participants with a major lower limb amputation (LLA) (at level of ankle and above).
- Included participants living in a lower-middle or low-income country according to the World Bank definition (World Bank, 2024).
- Included prosthetic limb users.
- Use of qualitative study design (ie, interviews, focus groups, grounded theory).
- Studies exploring views and experiences of life with a prosthetic limb.
- Presenting first-person accounts.

**Exclusion criteria**

- Included participants with upper limb or minor LLAs (ie, toes or partial foot) or studies that combined these populations with major LLAs.
- Included those not using a prosthetic limb or studies that combine these populations with limb wearers.
- Studies exploring prosthetic service provision.

### Critical appraisal

While the role of critical appraisal in qualitative synthesis is debated,^16 17^ its use is increasingly common. This review used the Critical Appraisal Skills Programme (CASP) tool^20^ to assess study quality and contextualise findings. Four included studies were independently appraised by two reviewers (CJF and CO), with any differences resolved through discussion. One study was appraised solely by CJF as CO was a coauthor of the primary study. CASP responses were scored from 1 (yes) to 3 (no), with no exclusions made—studies were ranked by quality to retain potentially valuable insights. Completed CASP checklists for included qualitative studies are provided in onlinesupplemental files S1S5.

### Data extraction

Data extraction was completed in two stages by CJF. In stage one, a structured form captured study details (aim, design, sample size, data collection method and LMIC setting), along with participant characteristics such as sex, cause and level of amputation and age range where available. In stage two, qualitative data were extracted from the results sections, including participant quotes and author interpretations supported by first-person quotes. CJF conducted the extraction and analysis using NVivo (QSR International, Melbourne, Australia).

### Analysis

Descriptive statistics summarised study designs and sample characteristics. Study findings were analysed using reflexive thematic analysis, as described by Braun and Clarke.^21^ This approach was selected rather than a thematic synthesis as the review addressed a different analytical question from those explored in the primary studies, leading to an interpretive re-analysis of the data, rather than the aggregative, line-by-line translation of typical thematic synthesis.^22^ As recommended in ENTREQ, coding was conducted within and across studies to generate themes that captured patterned meanings relevant to LMIC contexts. Table 3 outlines the analytical stages and contributor roles.

**Table 3:** Description of reflexive thematic analysis process

Phase	Description of process
Familiarisation	The results sections of the included studies were read and reread to increase familiarity with the data (CJF).
Coding	Open, line-by-line coding of the data was performed separately by the lead author (CJF). Extracts of text were coded in as many ways as needed. A reflective journal was completed throughout the analysis process to encourage awareness of the researcher’s own views and assumptions (CJF)
Generating initial themes	Codes and coded data were examined (CJF and CO), similarities and overlap were identified between codes and potential patterns relevant to the research question were created (CJF and CO)
Reviewing and developing new themes	A visual map of initial themes was created and compared (CJF and CO). All results sections were re-read and the fit of initial themes reviewed in relation to the full data set and coded data (CJF). This process was then repeated by members of the research team (CO).
Refining, defining and naming new themes	The set of themes was then reviewed and refined. Themes were collapsed or expanded to present coherent patterns within the data (CJF). The research team reviewed the themes to ensure they accurately captured meanings relevant to the research question and to facilitate reflection on researcher assumptions (CJF, CO and MD-H).
Writing up	Writing the report also acted as part of the process of refining and defining themes. Appropriate examples of extracts from the data were selected to represent each theme by CJF. A final report was produced by CJF, CO and MD-H.

### Research team and reflexivity

Contextual information about the research team has been presented here to enable readers to assess any influence our background and experience may have had on the research.^23^ CJF is a physiotherapist and qualitative researcher with experience in rehabilitation across both UK and LMIC contexts. CO is a UK-based consultant clinical academic physiotherapist with over 20 years’ experience in prosthetic rehabilitation clinical practice, and 15 years of experience in research. MD-H is a professor of health psychology with over 25 years of experience undertaking qualitative research with people following limb loss and complementary areas of rehabilitation.

Reflexive note keeping was undertaken by CJF throughout the review process and discussed regularly with the research team (CO and MD-H), in order to reflect on the impact of different perspectives and assumptions on analysis and interpretation of study data.

## Results

### Summary of included studies

**Figure 1:**
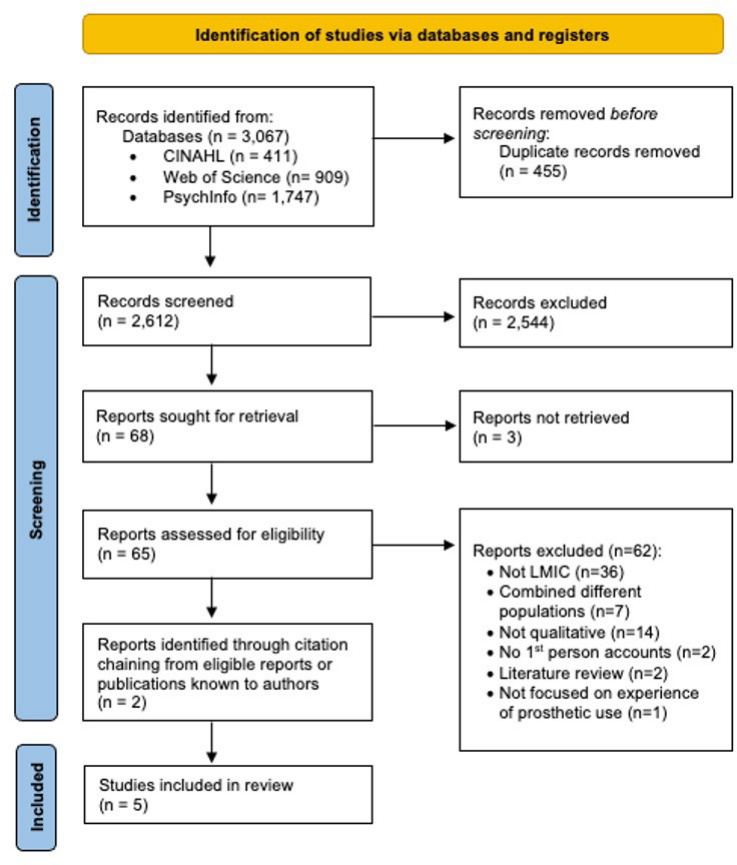
PRISMA flow diagram of study identification and selection. Flow diagram showing the identification, screening, eligibility assessment and inclusion of studies in the systematic review. Records were identified through database searches and citation chaining, with duplicates removed prior to screening. Reasons for exclusion at the full-text stage are detailed. Five studies met the inclusion criteria and were included in the final synthesis. The diagram is reported in accordance with PRISMA guidelines. LMIC, low- and middle-income country; PRISMA, Preferred Reporting Items for Systematic Reviews and Meta-Analyses.

The search yielded 3067 records. After removing duplicates and screening, 65 articles underwent full-text review. Three studies could not be retrieved in full text, despite exhaustive attempts, including contacting authors and searching alternative sources. Three studies met the inclusion criteria from the database search, and two further eligible studies were identified through author contact and citation chaining, resulting in a total of five included studies (figure 1).

The five included studies explored the experiences of 55 participants, 14 of whom were female (26%). Causes of amputation were trauma (n=41, 73%), diabetes (n=10, 18%), leprosy (n=2, 4%) and cancer (n=2, 4%). Amputation level was reported in four studies (n=45, 82%): transtibial level (n=29, 53%), transfemoral (n=14, 25%) and knee disarticulation (n=1, 2%), and one case of bilateral limb loss (2%). Participant ages ranged between 23 and 64 years. Study aims and sample characteristics are described in table 4.

**Table 4:** Summary of study aim and sample characteristics from papers included in the qualitative synthesis

Study	Aim	Location	Sample size (n)	Sample characteristics
Järnhammer *et al*^12^	To explore experiences of persons in Nepal using lower limb prostheses, in relation to the five articles of the CRPD, which consider mobility, education, including vocational training, health, rehabilitation and work and employment	Nepal	16	6 females. Level: 11 TTAs, 4 TFAs, 1 knee disarticulation. Cause: 3 civil wars, 6 traffic accidents, 2 leprosy, 2 cancer, 2 fall accidents, 1 snake bite. Living area: 4 rural, 2 urban, 4 hilly rural, 2 hilly urban, 1 plain rural, 3 plain urban Employment: 4 irregular, 6 no income, 6 regular. Vocational training: 8 yes
Mattick *et al*^13^	To explore the factors influencing the motivation of lower limb amputees engaging in prosthesis services in Mombasa, Kenya	Kenya	10	2 females. Age range 25–60. Time since amp: 2–20 years. Cause: 3 wound+diabetes, 3 workplace accidents, 2 car accidents, 1 playing football, 1 bicycle vs truck RTA. Distance from nearest prosthesis clinic: 4 km–150km
Donovan-Hall *et al*^24^ (preprint)	To understand the lived experiences of individuals currently using prosthetic services in Cambodia and explore views on the potential to trial planned technologies	Cambodia	12	1 female. Age range: 23–62. Level: 8 TFA, 4 TTA. Cause: 1 machinery accident, 4 traffic accidents, 3 land mines, 1 snake bite, 2 war accidents, 1 fall. Employment: 3 unemployed, 3 farmers, 1 housekeeping/cook, 1 factory worker, 2 street vendors, 1 construction, 1 gas attendant
Stuckey *et al*^26^	To explore the lived experience of people living in Bangladesh following LLA and prosthetic rehabilitation to understand the facilitators and barriers to work participation	Bangladesh	10	3 females. Age range: 24–63. Level: 7 TTAs, 2 TFAs, 1 bilateral TFA. Cause: 4 RTAs, 1 burn, 4 work accidents, 1 diabetes. Employment: 2 unemployed Vocational training: 4 yes
Lang and Svensk^25^	To describe activity and participation at home and in the community for individuals who have received a lower limb prosthesis	Kiribati	7	2 females. Age range: 47–64. Level: all TTAs. Cause: 7 diabetes

CRPD, Convention on the Rights of Persons with Disabilities; LLA, lower limb amputation; RTA, road traffic accident; TFA, transfemoral amputation; TTA, transtibial amputation.

The studies included 55 participants (14 females, aged 23–64) from Nepal, Kenya, Cambodia, Bangladesh and Kiribati using lower limb prostheses. Amputation causes include accidents, war, land mines, diabetes, burns and snake bites. Most had transtibial or transfemoral amputations, with some knee disarticulations and bilateral cases living in rural and urban areas, often far from prosthetic clinics. Employment varied, including farming, construction, factory work, unemployment and irregular income. Information on time since amputation and duration of prosthesis has not been presented here as data were reported inconsistently across the studies, with only one study providing extractable data.^12^

### Methodological quality of included studies

All included studies clearly stated their aims and used appropriate methods. However, only two discussed recruitment strategy and researcher–participant relationships, with one omitting this entirely. Reflexivity is vital, especially in LMIC settings, where power dynamics and cultural context can shape findings.^17^ Recruitment approaches are important because they influence who is represented, whose voices may be excluded and how participants engage with researchers. CASP scores ranged from 9 to 27 (lower=higher quality); studies contributing to this synthesis scored between 9 and 14 (online supplemental table T1).

### Themes

**Figure 2:**
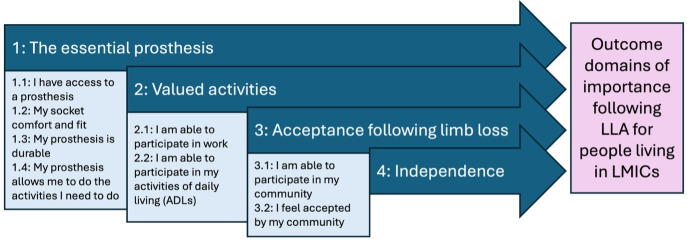
Conceptual framework of outcome domains of importance following lower limb amputation in LMICs. This figure illustrates the four overarching outcome domains identified from the synthesis of qualitative evidence: (1) the essential prosthesis, (2) valued activities, (3) acceptance following limb loss and (4) independence. Subdomains reflect key aspects within each domain, including access, comfort and durability of the prosthesis; participation in work and activities of daily living; community participation and social acceptance; and functional independence. Together, these domains represent outcome areas prioritised by people living with lower limb amputation in LMICs. LLA, lower limb amputation; LMICs, low- and middle-income countries.

Thematic analysis of the five studies identified four key themes reflecting outcome domains important to people with LLA in LMICs: (1) The importance of a prosthesis, (2) valued activities, (3) acceptance following limb loss and (4) independence. Figure 2 outlines the themes and subthemes, which are explored below with supporting quotations.

#### Theme 1: the importance of a prosthesis

This theme explores the role of prosthesis use in recovery after LLA in LMICs. Key outcome domains included socket comfort, prosthesis durability and functional suitability, discussed further in the subthemes below.

##### Subtheme 1.1: I have access to a prosthesis

All included studies highlighted the critical role of prosthesis provision in recovery for people with LLA in LMICs. Access to a prosthesis was universally viewed as essential to daily life,^1213 24^,^26^ with many participants describing it as life-changing: 

I use it all the time and it has helped me. I don’t think I can live without it. (Rany, Client, Lines 80–81)^24^

One participant highlighted the severe impact of lacking prosthetic access:

If I hadn’t that leg I don’t know where I would be and maybe I would be on the road begging but I am not begging and that’s why I am thankful that I am continuing with my life well. (Hamisi)^13^

##### Subtheme 1.2: my socket comfort and fit

In four studies,^1324^,^26^ participants emphasised that poor socket fit and comfort significantly affected prosthesis use:

There is not any problem but it is very loose when we work hard and walk it will shrink and become loose when we tie it, the leg will be swollen and it will be tight again for that we will take it off if hurting or affect anything it’s just hard to work when it is loose. If there is an inflammation, it will be painful. (Sarik, Client, Lines 69–72)^24^

Physically demanding jobs often caused socket discomfort, limiting participants’ ability to work as needed:

The owner asked me to use the prosthesis in the workplace too. But I cannot keep wearing it for more than four hours. So he said to use it for four hours. If the prosthesis fitted the stump a little bit better, I could continue to wear it for more hours and work faster than now. (Kamal)^26^

Some participants viewed perseverance and self-determination as key to coping with socket discomfort, which they felt improved over time:

[The prosthesis] was burning me…the rubber was very tight and walking was a problem…with time the skin became used after the swell and healing and it became comfortable. (Juma)^13^

##### Subtheme 1.3: my prosthesis is durable

Participants in the Nepal and Cambodia studies^12 24^ stressed the need for durable prostheses due to high use in physically demanding roles. Many used their prostheses until they were worn out, only seeking support once the device became unusable:

If it is hard to walk only then I only come to the rehabilitation centre. If it can be walk with I will not come. If as the appointment unless the foot is broken about 2 year or so I will come to receive the service and changing new foot is the most common because I walk a lot. (Sarik, Client, Lines 82–84)^24^

##### Subtheme 1.4: my prosthesis allows me to do the activities I need to do

While participants across all studies^1213 24^,^26^ valued their prostheses, many stressed the need for devices that supported daily activities like squatting, walking on uneven terrain or using in water. This was often highlighted when their prosthesis failed to meet these needs:

I can’t do anything when squatting, I need to be standing. But it is not possible to place firewood into the burner while standing. The problem is similar with harvesting activities… and I face difficulty while washing clothes because I can’t wet the prosthetic limb. I can’t use the prosthetic limb when saying prayers, ablutions, sleeping and taking a shower. (Rina)^26^

Environmental challenges also limited prosthetic use:

My house is distant from my shop, about half a kilometre…through bushes and trees. So, at night I cannot travel back up and down a muddy road. I might fall, so with this fear I go back earlier. (Ronnie)^26^

### Theme 2: valued activities

The second theme highlights the importance of engaging in valued activities for people with LLA in LMICs. The prosthesis was often seen as essential for this, particularly for returning to work and performing daily tasks.

#### Subtheme 2.1: I am able to participate in work

Paid employment was the most frequently mentioned and valued activity, discussed in all five studies:

I think if we do not wear any leg, we cannot work at all but if we wear it, we can go anywhere, we can earn money to support children and the wife and the family. (Soy, Client, 58–59).^24^

Work was seen as essential for restoring social identity and sustaining the family’s livelihood, often driven by financial hardship or caregiving responsibilities:

Everyone in my family depends on me … There are four people in my family. I need to take care of all of them, like food, and everything. I am bearing all the responsibilities for the income. (Mostafa^26^

For some participants, returning to work appeared to be a matter of pride:

For now, I can work and get my own 500ksh without asking to be given by anybody, I am able to do my own work without depending on anybody… [I can] buy my children sugar for tea to be like other children. (Juma)^13^

Work was also described as a key source of well-being, with both positive and negative aspects highlighted:

I enjoy my work very much. I feel free when I chat with those around me. I am well because of my work. (Himel)^26^

I go outside and sit on my own. I feel sorrow and I feel I’m not like others […]I’m unemployed so I feel I’m not able to do anything […]. (Male B, living in rural area)^12^

Despite the importance of returning to work, many participants faced barriers, including discrimination rooted in perceptions of physical incapacity:

I think I’ve been unemployed because of my disability, but I think […]I can do easy work. But people won’t hire me because of my disability […] I think disability is the main reason behind my unemployment. (Male G, living in hilly rural area)^12^

#### Subtheme 2.2: I am able to participate in my activities of daily living

All five studies highlighted participation in activities of daily living (ADLs)—such as personal care, toileting and domestic tasks—as a key outcome. Successfully completing these activities, often with a prosthesis, fostered independence and personal satisfaction:

I can take myself to the shop without any help, unlike before when I used to depend on people to help me, I can go by myself to the toilet without asking for help. (Mohamed)^13^

It is not possible to do this type of work without the prosthesis …I can take care of myself now, I couldn’t even go to the wash room when I didn’t have it. (Rina^26^

Participants described adapting to challenges in order to engage in valued activities:

I want to overcome the physical limitations by thinking and planning. If I can’t take the bus, I take the rickshaw instead. As I have difficulty getting up and down, I wear pants instead of a loin cloth. I worked these things out for myself … I faced a problem with the toilet. There is no high commode in the village, but I carry a rubber tire tube for sitting. (Mostafa)^26^

Situations where people were unable to take part in their daily activities were described in a negative way.

I want to do like I used to…go on walks…but I can’t do that because sometimes…I get tired…pain sometimes…^25^

Some participants reported that being unable to perform daily activities negatively affected their mental health, family life and finances:

Our kids are scared, because if I don’t continue my domestic responsibilities their father might get married for a second time. (Rina)^26^

### Theme 3: acceptance following limb loss

Beyond daily activities, participants highlighted the importance of community participation. This was closely tied to acceptance after limb loss, shaping self-perception and how others viewed their disability. Acceptance emerged as a key recovery outcome, explored here in two subthemes.

#### Subtheme 3.1: I am able to participate in my community

Participants expressed a strong desire to feel ‘part of’ rather than ‘apart from’ their community, often through involvement in activities like church or community roles.

I always pray… During the Sundays, I go and join the church function. I never miss… a function.’ ‘Involve in…my working place activity…I just join the community…’ ‘…I like to be involved in a lot of things…I have a really active wife…I… work with her…’^25^

For this participant, their occupation was a source of pride and cultural identity, reinforcing their sense of belonging within a work-based community:

In our culture our main food is fishing…most of the men will do fishing… ‘I stop fishing…I really want to do fishing and build…my own house…but I can’t’ ‘…just like fishing…that’s the main thing I want’.^25^

‘Essential’ prosthesis use was the key enabler of community participation:

‘Now…when I want to go to neighbours I just put on my leg and go.’ ‘…I walk with my prosthesis even in the night… if it’s too far I catch the public bus…’ ‘I use my prosthesis to go and play the bingo game in the other places. It’s quite far but I can walk…even if it’s twelve at midnight.^25^

#### Subtheme 3.2: I feel accepted by my community

Across all five studies, prosthesis use and community participation were closely tied to participants’ acceptance and adjustment to life after amputation—an important outcome. This was described both positively and negatively. Some participants reported stigma and feeling ‘unlike’ others, leading to feelings of inadequacy:

I used to see as if my life was over, moving in crutches, people look at you and say that you are hurt or lame (Hamisi)^13^

Others shared that socialising reduced their sense of disability and improved well-being and quality of life.

Involving with…other people help me to build up the confidence and like self-esteem…it also help me to reduce…that I already disabled…Involving with other people give me more encouragement. I just want to enjoy my life. …if I want to visit friends I can go because I have the leg… if I want to join… I just go…^25^

Fulfilling valued community roles—such as positions of authority—was seen as supporting acceptance, identity and recovery.

Where I am, people call me “boss” and I am very thankful, and when you see me you will not know [about the amputation], and you will just know I am a boss of a certain area (Hamisi)^13^

Community support during times of need also fostered acceptance and strengthened belonging.

It’s just our families and neighbours [that support] when I don’t have prosthetic leg to use, they came to help, help to carry. They don’t discriminate at all, they even help to encourage… (Sarik. Client, Lines 91–93).^24^

Peer support was widely seen as informative and motivating, helping to promote acceptance and adjustment.

After coming here to the hospital, outside my community, I saw more people like me and seeing the people have the same problem and by talking with them and sharing all the problems I got a feeling of strong encouragement to do something more in my life. (Male F, living in rural area)^12^

### Theme 4: independence

All five studies identified independence as a vital aspect of recovery, closely linked to the previous themes. Prosthesis use enabled physical independence, facilitating participation in valued activities like work and reducing their reliance on others—enhancing self-esteem and acceptance. Fear of unmet basic needs also motivated participants to pursue independence.

I think if we do not wear any leg, we cannot work at all, but if we wear it, we can go anywhere, we can earn money to support children and the wife and the family. (Soy, Client, 58–59).^24^

Participants also described how losing independence affected both themselves and their families, citing financial strain and feeling like a burden.

I’m feeling guilty because my father and my brother are having problems to meet my expenses, so if they are in trouble, I feel guilty because I created the problem for them. (Male D, living in urban area)^12^

Independence and control over one’s future were seen as key to recovery and well-being:

There is no better food than that from one’s own hand. It is a psychological satisfaction for myself to work. Being dependent on others, even for food, cannot satisfy anyone. (Rina)^26^

## Discussion

To our knowledge, this is the first study to investigate outcome domains valued by individuals undergoing lower limb prosthetic rehabilitation in LMICs. In the absence of direct empirical research, we conducted a systematic review and qualitative synthesis of studies capturing lived experiences across diverse LMIC contexts.

The synthesis included five studies describing the experiences of 55 adults living with LLA across Nepal, Cambodia, Bangladesh, Kiribati and Kenya. Participants (41 men and 14 women; aged between 23 and 64) had varied amputation aetiologies. Despite this diversity, the small number of studies and countries represented limits the evidence base, especially given the millions living with limb loss across LMICs.^10^ While not comprehensive, this review is the first to explore valued outcomes in these settings and offers important, original insights. Key domains identified include: (1) access to a comfortable, durable prosthesis, (2) returning to valued activities (eg, work, ADLs), (3) community participation that promotes acceptance and (4) independence.

While no prior studies have explored outcome domains specifically in LMICs, research from high-income settings has identified similar priorities, such as prosthesis comfort,^2 27^ participation in valued activities,^2^,^4^ psychosocial adjustment^2^,^46^ and independence.^2^,^4^ These parallels suggest some aspects of recovery may be universal, but key differences emerged in LMIC narratives. This was notably the emphasis on returning to work and prosthesis durability, less prominent in high-income studies, suggesting greater relevance in low-resource settings. Conversely, issues like pain management,^2 6 27^ falls,^2 6 27^ equipment^4 6^ and psychological rehabilitation^2 6^—commonly noted in high-income contexts—were not discussed in the LMIC studies reviewed. It is possible that cultural and contextual factors may have influenced how participants reported pain, discomfort and mobility difficulties. Studies in South Asian and other cultural settings describe norms of endurance and stoicism that shape how openly individuals express pain or functional challenges.^28 29^ Expectations regarding falls and mobility may similarly reflect environmental realities in many LMIC contexts, where uneven terrain, limited pavements and variable household infrastructure make falls more commonplace. These environmental conditions were evident in the study from Nepal, where participants described hilly terrain and irregular walking surfaces as everyday challenges when using a prosthesis.^12^ Wider evidence also shows that hazardous environments and poor infrastructure contribute significantly to mobility risk in LMICs.^30^ These cultural and infrastructure influences may help explain the paucity of data surrounding these domains.

The absence of certain outcome domains in LMIC studies, such as pain and falls,^2 3^ raises questions about whether these issues are genuinely less relevant or simply underexplored. This gap underscores the limited understanding of prosthetic rehabilitation in these settings, where research has largely focused on access challenges,^22 23^ which affect many individuals undergoing LLA according to WHO estimates.^12^ A recent review by Ghai *et al*^31^ examined the reporting of rehabilitation outcomes in traumatic lower limb amputation studies and highlighted the importance of variables such as treatment pathways and duration of prosthetic rehabilitation in understanding recovery trajectories. These factors may influence outcome priorities; however, the LMIC studies included in our review did not consistently report rehabilitation timelines, types of prosthetic interventions received or duration of prosthesis use. As a result, these variables could not be synthesised. This gap reflects broader challenges in LMIC reporting standards and underscores the need for future research to document rehabilitation processes in greater detail. Equally, understanding the experiences and outcomes of those who do receive a prosthesis is crucial to ensure services are fit for purpose, an important focus for future research.

As expected, access to a prosthesis emerged as a key outcome across the included studies, with participants repeatedly emphasising its essential role. This aligns with broader literature highlighting the importance of prosthetic and assistive technology for individuals with LLA in LMICs, where access is often limited by inadequate facilities, resources and infrastructure.^32^,^37^

In addition to issues of access, participants highlighted challenges related to prosthesis use, particularly socket discomfort, which was exacerbated by prolonged wear or walking on uneven or wet terrain. While limited literature explores the lived experience of prosthetic socket use in any setting, there is a notable gap in LMIC-specific research on postoperative or chronic pain.^38 39^ Evidence from HICs documents the prevalence of socket-related complications, including pain, skin breakdown and infection.^40 41^

In addition to socket comfort, participants emphasised the need for durable prostheses capable of withstanding frequent use in challenging environments. Some reported wearing prostheses until they were no longer functional, either due to financial constraints or limited access to prosthetic services, findings supported by existing literature,^42^,^44^ which documents ongoing device use despite a need for repair or replacement. A study in Cambodia^45^ found that prostheses were typically replaced every 2 years, with repair needs influenced by factors such as gender, usage, weight and age. The International Committee of the Red Cross (ICRC) has long promoted context-appropriate prosthetic technologies designed to be durable, cost-effective and compatible with local climates.^46 47^ While high levels of physical function and quality of life have been reported in ICRC-rehabilitated users in Myanmar,^48^ durability was not specifically assessed. This evidentiary gap underscores the need to investigate user experiences with socket comfort and device longevity in LMICs to inform both clinical practice and prosthetic design.

Participants across the Kiribati, Nepalese and Kenyan studies described prostheses as vital for valued activities such as fishing, walking and climbing, yet many reported they were poorly adapted to specific functional needs. Tasks like squatting, kneeling to pray and traversing uneven terrain were described as difficult, limiting participation in culturally meaningful activities. This dissatisfaction reflects broader literature on prosthesis ‘satisfaction with use’, often framed around fit, comfort and appearance,^32 49^ but also highlights how existing research tends to focus narrowly on ambulation and transfers while overlooking contextually relevant tasks in LMICs. Although componentry details were rarely provided, prosthetic options in these settings remain limited, with ICRC technologies prioritising cost-efficiency and durability over functionality. A deeper understanding of how individuals in low-resource settings use their prostheses—combined with user satisfaction data—could inform the development of context-appropriate solutions and rehabilitation priorities. The WHO Standards for Prosthetics and Orthotics^50^ emphasise the importance of people-centred, needs-responsive prosthetic services, recognising that access to prostheses supporting essential daily or work movements is central to employment, the most frequently prioritised outcome across participants.

Across all five studies, participation in work emerged as a critical outcome domain—framed not only as means of financial survival but also as a pathway to social inclusion, personal fulfilment and identity reconstruction. This emphasis reflects wider literature on disability in LMICs, where employment is consistently linked to independence, dignity and reduced stigma.^51 52^ While return to work is mentioned in studies from high-income contexts,^32 53 54^ it appears less prominent—likely due to stronger social welfare systems that mitigate the economic impact of disability. In LMICs where such safety nets are limited or absent, work becomes essential for livelihood, with failure to regain employment often associated with poverty, marginalisation and psychosocial decline. Participants also described how employment enhanced their standing within the community, shifting perceptions away from visible impairment toward personal value and contribution. This experience links closely to broader constructs of independence and self-acceptance, as individuals expressed a deep desire to feel useful, purposeful and needed. While research has shown that having a sense of purpose supports emotional resilience and recovery,^55 56^ little is known about this in the context of LLA, particularly in LMICs. Notably, anxieties around occupational adaptation and social reintegration appear especially pronounced among those who are primary earners, with some evidence suggesting these concerns can hinder rehabilitation progress.^57^

Engagement in work and community life appeared to facilitate psychosocial adjustment, acting as a pathway through which individuals accepted their new reality and re-established their roles within society. While the process of psychosocial adjustment following amputation and prosthesis use is well documented in HICs,^26 58^,^60^ significantly less is known about how individuals in LMICs navigate this transition. In resource-rich contexts, access to psychological rehabilitation services may support this process, but in LMICs, the availability and nature of such support is often limited. Consequently, the mechanisms through which people adjust, the factors influencing their adaptation and the types of interventions that may be most effective remain unclear and warrant further investigation.

### Limitations

This review has several limitations, the most apparent being the inclusion of only five eligible studies, reflecting the experiences of just 55 people with limb loss, including only 14 women. The small size and gender imbalance of the evidence base highlight the paucity of research on recovery following LLA in LMICs and underscores the urgent need for further investigation. These limitations constrain the transferability of the findings and limit the extent to which they can inform our understanding of valued outcome domains in these settings. As such, the findings should be viewed as initial insights that provide a foundation for more robust future research.

The review adopted a broad search strategy to identify all qualitative research involving lower limb prosthesis users, given the absence of studies explicitly examining outcome domains of importance in LMICs. Consequently, the included studies explored a diverse range of phenomena, necessitating an exploratory synthesis more typical of primary qualitative research. This required re-analysis of findings through a different analytical lens than that applied in the primary studies, introducing the possibility of reinterpretation or meaning shift.

Studies involving mixed populations, such as those combining upper and lower limb amputees or prosthetic and orthotic users, were excluded where first-person data could not be separated. Many studies did not specify whether quotations originated from people with upper or lower limb loss, which differ substantially in rehabilitation pathways, prosthetic provision, functional impact and psychosocial adjustment.^51^ Although this may have resulted in the omission of potentially relevant insights, including these data risked generating themes not applicable to LLA specifically.

By synthesising LMIC data into a single analysis, this review combined perspectives from diverse political, economic and cultural contexts. While this was necessary due to the limited evidence base, it may have obscured context-specific factors shaping outcome priorities. In addition, the included studies varied in methodological quality. Only two adequately addressed the researcher–participant relationship, and none included researcher positionality statements, an important omission given the potential for power imbalances and cultural sensitivity in LMIC settings.^52^ One non-peer-reviewed study was included due to the scarcity of available research.

Important variables which may have impacted outcome domains of importance to patients, such as time since amputation, duration of prosthesis use and details of rehabilitation pathways, were inconsistently reported across the included studies and therefore could not be synthesised. Furthermore, all included studies recruited participants from prosthetic clinics or rehabilitation services, suggesting the review may only represent the views of care-seeking individuals.

Several methodological constraints warrant consideration. No protocol was registered for this review, although protocol registration is less common for qualitative evidence syntheses. The search was undertaken in April 2024 and any studies published thereafter were not captured. PubMed was not included in the database search strategy as databases with more complete indexing for qualitative studies were selected. Non-English language studies were excluded; however, only a single non-English language study was identified which was not undertaken within an LMIC setting. Finally, only one reviewer undertook 80% of screening and selection and all data extraction.

## Conclusions

Despite limited research, this review offers early insights into outcome domains of importance after receiving a prosthesis in LMICs. Access to prosthesis is vital, enabling return to work—essential for survival—and participation in society. Independence, acceptance and adjustment appear closely tied to these outcomes. Yet issues with socket comfort, fit and durability affect satisfaction with prosthesis function. The lack of evidence on lived experience, particularly around psychosocial adjustment, underscores the need for further research. Given the diversity within LMICs, exploring these domains across varied settings and cultures is essential.

## Supplementary material

10.1136/bmjopen-2025-109817 online supplemental file 1

10.1136/bmjopen-2025-109817 online supplemental file 2

10.1136/bmjopen-2025-109817 online supplemental file 3

10.1136/bmjopen-2025-109817 online supplemental file 4

10.1136/bmjopen-2025-109817 online supplemental file 5

10.1136/bmjopen-2025-109817 online supplemental table 1

## Data Availability

All data relevant to the study are included in the article or uploaded as supplementary information.
