# Supplementary material for: Exploring meaningful outcome domains of recovery following lower limb amputation (LLA) and prosthetic rehabilitation in low- and middle-income (LMIC) settings: a qualitative systematic review
Source: BMJ Open. 2026 Jan 28;16(1):e109817. doi: 10.1136/bmjopen-2025-109817 (PMC12853489; doi:10.1136/bmjopen-2025-109817)
Supplement: online supplemental file 1 [file bmjopen-16-1-s001.docx]

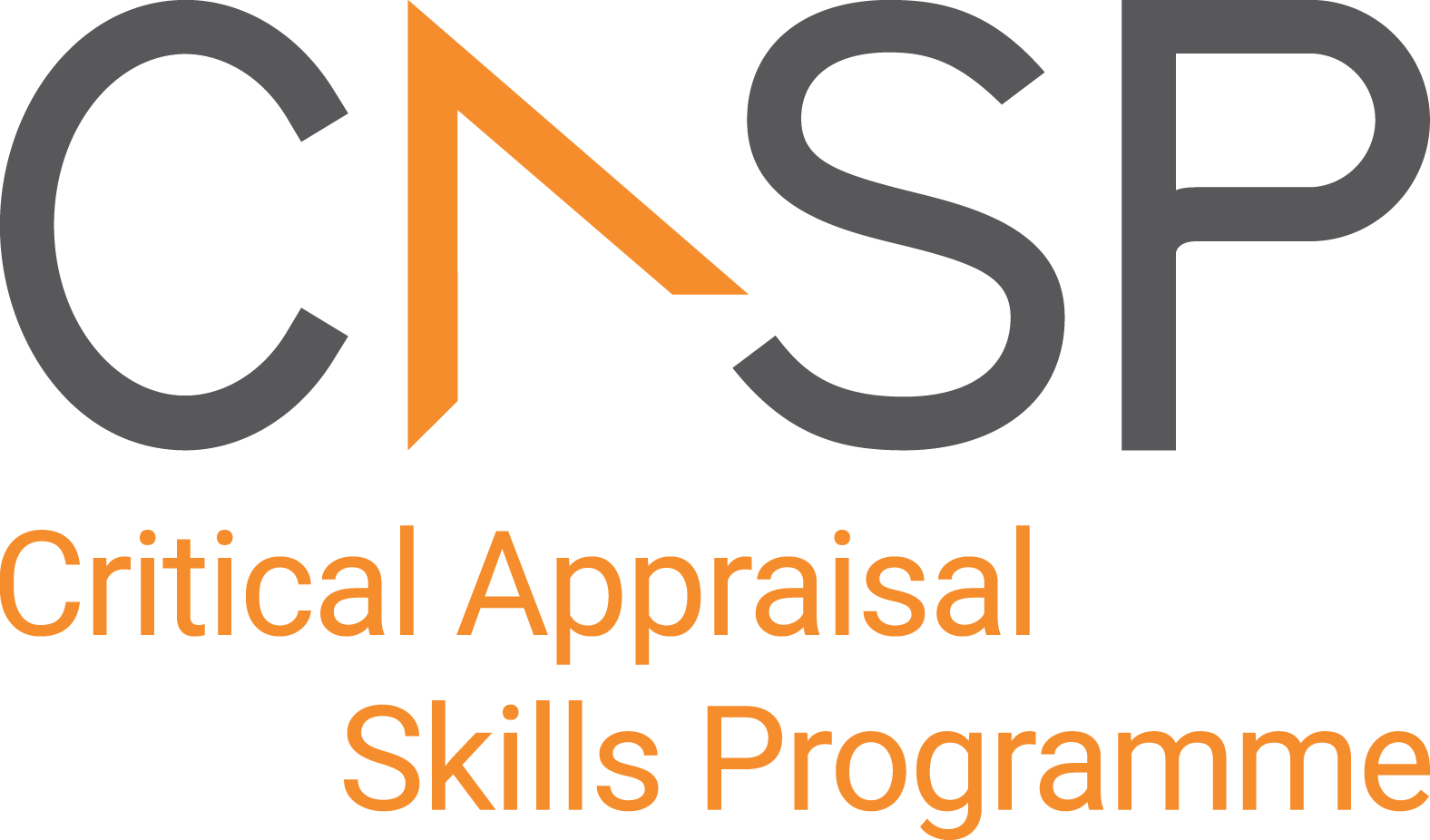
CASP Checklist:

For Qualitative Research

| **Paper Title:** | Experiences of lower limb prosthesis users  in Kenya: a qualitative study to understand  motivation to use and satisfaction with prosthetic  outcomes |
| --- | --- |
| **Author:** | Kate Mattick, Ben Oldfrey, Maggie Donovan-Hall, Grace Magomere, Joseph Gakunga & Catherine Holloway |
| **Web Link:** | https://doi.org/10.1080/09638288.2022.2152875 |
| **Appraisal Date:** | May 2024 |

During critical appraisal, never make assumptions about what the researchers have done. If it is not possible to tell, use the “Can’t tell” response box. If you can’t tell, at best it means the researchers have not been explicit or transparent, but at worst it could mean the researchers have not undertaken a particular task or process. Once you’ve finished the critical appraisal, if there are a large number of “Can’t tell” responses, consider whether the findings of the study are trustworthy and interpret the results with caution.

| **Section A Are the results valid?** | |
| --- | --- |
| 1. Was there a clear statement of the aims of the research? | Yes  No  Can’t Tell |
| *CONSIDER:*   - *what was the goal of the research?* - *why was it thought important?* - *its relevance*   Yes, the paper clearly states its aim: to explore the factors influencing motivation of lower-limb amputees engaging with prosthesis services in Mombasa, Kenya | |
| 1. Is a qualitative methodology appropriate? | Yes  No  Can’t Tell |
| *CONSIDER:*   - *If the research seeks to interpret or illuminate the actions and/or subjective experiences of research participants* - *Is qualitative research the right methodology for addressing the research goal?*   The study seeks to understand personal experiences, motivations and perceptions of prosthesis users: inherently subjective phenomena. Therefore, qualitative methodology is appropriate | |
| 1. Was the research design appropriate to address the aims of the research? | Yes  No  Can’t Tell |
| *CONSIDER:*   - *if the researcher has justified the research design (e.g., have they discussed how they decided which method to use)*   The authors justify using semi-structured interviews and inductive thematic analysis to explore lived experience and motivation aligning with the aim of understanding subjective experiences in context | |
| 1. Was the recruitment strategy appropriate to the aims of the research? | Yes  No  Can’t Tell |
| *CONSIDER:*   - *If the researcher has explained how the participants were selected* - *If they explained why the participants they selected were the most appropriate to provide access to the type of knowledge sought by the study* - *If there are any discussions around recruitment (e.g. why some people chose not to take part)*   Participants were purposively recruited from Association for the Physically Disabled of Kenya (APDK). The authors acknowledge limitations e.g., only those engaged with ADPK and requiring mobile phone access | |
| 1. Was the data collected in a way that addressed the research issue? | Yes  No  Can’t Tell |
| *CONSIDER:*   - *If the setting for the data collection was justified* - *If it is clear how data were collected (e.g. focus group, semi-structured interview etc.)* - *If the researcher has justified the methods chosen* - *If the researcher has made the methods explicit (e.g. for interview method, is there an indication of how interviews are conducted, or did they use a topic guide)* - *If methods were modified during the study. If so, has the researcher explained how and why* - *If the form of data is clear (e.g. tape recordings, video material, notes etc.)* - *If the researcher has discussed saturation of data*   Data collection methods are explicitly described: semi-structured interviews via Microsoft Teams, using a culturally informed interview guide, with Swahili translation where appropriate. Audio-recording and transcription are clearly stated. Thematic saturation is discussed conceptually through sample-size rationale (10 participants) | |
| 1. Has the relationship between researcher and participants been adequately considered? | Yes  No  Can’t Tell |
| *CONSIDER:*   - *If the researcher critically examined their own role, potential bias and influence during (a) formulation of the research questions (b) data collection, including sample recruitment and choice of location* - *How the researcher responded to events during the study and whether they considered the implications of any changes in the research design*   The paper provides detailed reflexivity: JG was a clinician at APDK where recruitment could introduce bias. KM reflects on own physiotherapy background and assumption; steps to minimise bias are outlined (e.g., using non-clinical co-researchers, independence translator, audit trail, triangulation) | |
| **Section B: What are the results?** | |
| 1. Have ethical issues been taken into consideration? | Yes  No  Can’t Tell |
| *CONSIDER:*   - *If there are sufficient details of how the research was explained to participants for the reader to assess whether ethical standards were maintained* - *If the researcher has discussed issues raised by the study (e.g. issues around informed consent or confidentiality or how they have handled the effects of the study on the participants during and after the study)* - *If approval has been sought from the ethics committee*   Multiple ethics approvals were obtained (APDK, AIC Cure Hospital IRB, UCL EC) consent procedures are clearly described, and confidentiality considerations noted | |
| 1. Was the data analysis sufficiently rigorous? | Yes  No  Can’t Tell |
| *CONSIDER:*   - *If there is an in-depth description of the analysis process* - *If thematic analysis is used. If so, is it clear how the categories/themes were derived from the data* - *Whether the researcher explains how the data presented were selected from the original sample to demonstrate the analysis process* - *If sufficient data are presented to support the findings* - *To what extent contradictory data are taken into account* - *Whether the researcher critically examined their own role, potential bias and influence during analysis and selection of data for presentation*   The authors describe using Braun & Clarke’s six-phase thematic analysis. They provide a visual thematic map and present several quotes. Contradictions (such as differing attitudes to prosthesis aesthetics) are acknowledged. | |
| 1. Is there a clear statement of findings? | Yes  No  Can’t Tell |
| *CONSIDER:*   - *If the findings are explicit* - *If there is adequate discussion of the evidence both for and against the researcher’s arguments* - *If the researcher has discussed the credibility of their findings (e.g. triangulation, respondent validation, more than one analyst)* - *If the findings are discussed in relation to the original research question*   Five themes are clearly named, defined, and supported with participant quotations. Findings link explicitly back to the research questions and literature, and implications for rehabilitation and service design are discussed. | |
| **Section C: Will the results help locally?** | |
| 1. How valuable is the research? | Yes  No  Can’t Tell |
| *CONSIDER:*   - *If the researcher discusses the contribution the study makes to existing knowledge or understanding (e.g., do they consider the findings in relation to current practice or policy, or relevant research-based literature* - *If they identify new areas where research is necessary* - *If the researchers have discussed whether or how the findings can be transferred to other populations or considered other ways the research may be used*   The authors articulate contributions to knowledge: new insights into prosthesis motivation in an LMIC context, implications for clinician training, service design, stigma, device acceptance and peer support, identification of areas for future research (lost-to-follow-up, stigma, service decentralisation). Transferability issues are acknowledged. | |

| **APPRAISAL SUMMARY**: *List key points from your critical appraisal that need to be considered when assessing the validity of the results and their usefulness in decision-making.* | | |
| --- | --- | --- |
| **Positive/Methodologically sound** | **Negative/Relatively poor methodology** | **Unknowns** |
| **Clear aim and strong justification for qualitative design**  **Transparent recruitment process with acknowledged limitations**  **Culturally sensitive data collection, clear methods, and reflexive awareness of bias**  **Rigorous thematic analysis following established frameworks, supported by quotes**  **Ethical approval and procedures well described**  **Findings are coherent and linked to wider literature** | **Participants were only those actively engaged with APDK; those who dropped out, disengaged, or lacked phone access were excluded (potential positive bias)**  **Recruitment was opportunistic: the first 10 approached agreed – no exploration of refusals**  **Virtual interviews may have constrained rapport compared to in-person interviews**  **Limited demographic diversity (all transtibial amputees, only two women)** | **Depth of iterative changes to interview guide not detailed**  **Extent of data saturation not explicitly demonstrated, only implied through sample-size rationale** |

**Referencing recommendation:**

CASP recommends using the Harvard style referencing, which is an author/date method. Sources are cited within the body of your assignment by giving the name of the author(s) followed by the date of publication. All other details about the publication are given in the list of references or bibliography at the end.

Example:

*Critical Appraisal Skills Programme (2024). CASP (insert name of checklist i.e. systematic reviews with meta-analysis of randomised controlled trials (RCTs) Checklist.) [online] Available at: insert URL. Accessed: insert date accessed.*

**Creative Commons**

©CASP this work is licensed under the Creative Commons Attribution – Non-Commercial- Share A like. To view a copy of this licence, visit <https://creativecommons.org/licenses/by-nc-sa/4.0/>

**
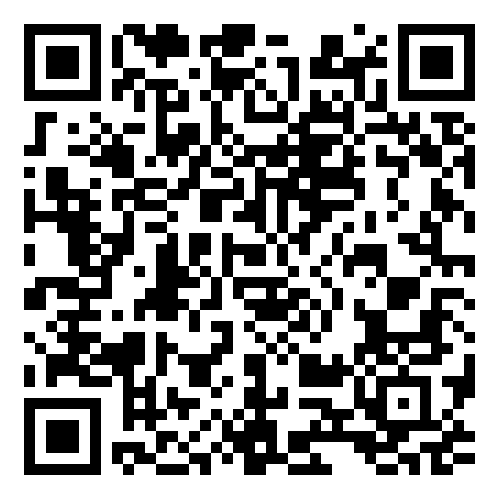
Need further training on evidence-based decision making?** Our online training courses are helpful for healthcare educational researchers and any other learners who:

- Need to critically appraise and stay abreast of the healthcare research literature as part of their clinical duties.
- Are considering carrying out research & developing their own research projects.
- Make decisions in their role, whether that be policy making or patient facing.

**Benefits of CASP Training:**

- Affordable – courses start from as little as £6
- Professional training – leading experts in critical appraisal training
- Self-directed study – complete each course in your own time
- 12 months access – revisit areas you aren’t sure of and revise
- CPD certification - after each completed module

Scan the QR code below or visit <https://casp-uk.net/critical-appraisal-online-training-courses/> for more information and to start learning more.
