# Supplementary material for: Exploring meaningful outcome domains of recovery following lower limb amputation (LLA) and prosthetic rehabilitation in low- and middle-income (LMIC) settings: a qualitative systematic review
Source: BMJ Open. 2026 Jan 28;16(1):e109817. doi: 10.1136/bmjopen-2025-109817 (PMC12853489; doi:10.1136/bmjopen-2025-109817)
Supplement: online supplemental file 2 [file bmjopen-16-1-s002.docx]

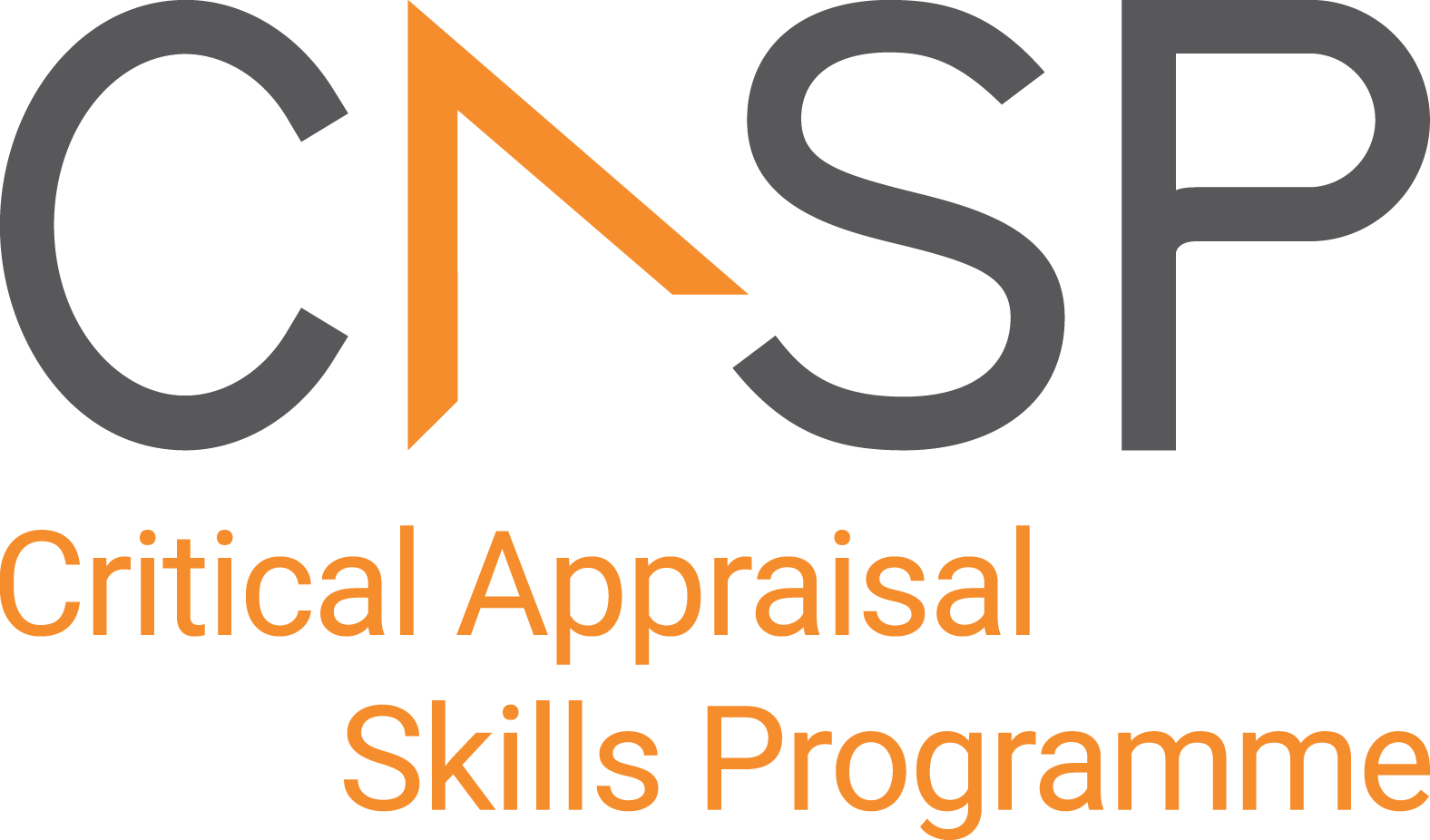
CASP Checklist:

For Qualitative Research

| **Paper Title:** | Exploring the experiences of individuals and their families using prosthetic devices and services in Cambodia following lower limb amputation |
| --- | --- |
| **Author:** | Maggie Donovan-Hall, Chantel Ostler, Ratana Sann, Cheryl D Metcalf, Peter Worsley, Sisary Kheng, Sam Simpson, Carson Harte, Alex Dickinson |
| **Web Link:** | Paper in process of being uploaded to preprint serve MedRxiv |
| **Appraisal Date:** | May 2024 |

During critical appraisal, never make assumptions about what the researchers have done. If it is not possible to tell, use the “Can’t tell” response box. If you can’t tell, at best it means the researchers have not been explicit or transparent, but at worst it could mean the researchers have not undertaken a particular task or process. Once you’ve finished the critical appraisal, if there are a large number of “Can’t tell” responses, consider whether the findings of the study are trustworthy and interpret the results with caution.

| **Section A Are the results valid?** | |
| --- | --- |
| 1. Was there a clear statement of the aims of the research? | Yes  No  Can’t Tell |
| *CONSIDER:*   - *what was the goal of the research?* - *why was it thought important?* - *its relevance*   The study has a clearly stated aim, to explore lived experiences of prosthetic users and their families in Cambodia and inform wider service and technology improvement | |
| 1. Is a qualitative methodology appropriate? | Yes  No  Can’t Tell |
| *CONSIDER:*   - *If the research seeks to interpret or illuminate the actions and/or subjective experiences of research participants* - *Is qualitative research the right methodology for addressing the research goal?*   The study sought to understand lived experiences, beliefs, and contextual issues, therefore qualitative design is appropriate | |
| 1. Was the research design appropriate to address the aims of the research? | Yes  No  Can’t Tell |
| *CONSIDER:*   - *if the researcher has justified the research design (e.g., have they discussed how they decided which method to use)*   Semi-structured interviews and reflexive thematic analysis are appropriate for exploring lived experiences and this was explicitly justified (stakeholder-led, flexible, co-produced, inductive) | |
| 1. Was the recruitment strategy appropriate to the aims of the research? | Yes  No  Can’t Tell |
| *CONSIDER:*   - *If the researcher has explained how the participants were selected* - *If they explained why the participants they selected were the most appropriate to provide access to the type of knowledge sought by the study* - *If there are any discussions around recruitment (e.g. why some people chose not to take part)*   Convenience sample was taken from three clinics, there were multiple recruitment routes (posters, clinician referral, telephone), consideration of literacy, and inclusion of family members present. | |
| 1. Was the data collected in a way that addressed the research issue? | Yes  No  Can’t Tell |
| *CONSIDER:*   - *If the setting for the data collection was justified* - *If it is clear how data were collected (e.g. focus group, semi-structured interview etc.)* - *If the researcher has justified the methods chosen* - *If the researcher has made the methods explicit (e.g. for interview method, is there an indication of how interviews are conducted, or did they use a topic guide)* - *If methods were modified during the study. If so, has the researcher explained how and why* - *If the form of data is clear (e.g. tape recordings, video material, notes etc.)* - *If the researcher has discussed saturation of data*   There was clear detail in the reporting of data collection. Semi-structured interviews undertaken in indigenous language, in multiple settings (clinics/homes), audio recordings, memos, translation and back-translation and co-designed interview guides. | |
| 1. Has the relationship between researcher and participants been adequately considered? | Yes  No  Can’t Tell |
| *CONSIDER:*   - *If the researcher critically examined their own role, potential bias and influence during (a) formulation of the research questions (b) data collection, including sample recruitment and choice of location* - *How the researcher responded to events during the study and whether they considered the implications of any changes in the research design*   Although the paper includes researcher background descriptions, mention of a reflexive diary, and team-based analysis including Cambodian partners, it does not provide detail on how researcher-participant power dynamics, social desirability bias, or interviewer influence were managed during interviews, especially considering that interviews sometimes occurred inside the clinics providing free prosthetics services, and local interviewers had inconsistent qualitative experience. | |
| **Section B: What are the results?** | |
| 1. Have ethical issues been taken into consideration? | Yes  No  Can’t Tell |
| *CONSIDER:*   - *If there are sufficient details of how the research was explained to participants for the reader to assess whether ethical standards were maintained* - *If the researcher has discussed issues raised by the study (e.g. issues around informed consent or confidentiality or how they have handled the effects of the study on the participants during and after the study)* - *If approval has been sought from the ethics committee*   Multiple ethics approvals sought, informed consent procedures described, 24-hour consideration time given, anonymity and confidentiality procedures explicitly stated. | |
| 1. Was the data analysis sufficiently rigorous? | Yes  No  Can’t Tell |
| *CONSIDER:*   - *If there is an in-depth description of the analysis process* - *If thematic analysis is used. If so, is it clear how the categories/themes were derived from the data* - *Whether the researcher explains how the data presented were selected from the original sample to demonstrate the analysis process* - *If sufficient data are presented to support the findings* - *To what extent contradictory data are taken into account* - *Whether the researcher critically examined their own role, potential bias and influence during analysis and selection of data for presentation*   The study included strong description and rigour through reflexive thematic analysis, with mentions of multi-stage coding, team-based analysis including Cambodian partners, verification in team meetings, and explicit coding to theme description | |
| 1. Is there a clear statement of findings? | Yes  No  Can’t Tell |
| *CONSIDER:*   - *If the findings are explicit* - *If there is adequate discussion of the evidence both for and against the researcher’s arguments* - *If the researcher has discussed the credibility of their findings (e.g. triangulation, respondent validation, more than one analyst)* - *If the findings are discussed in relation to the original research question*   There is a clear three-category structure, nine themes, extensive quotations, explicitly interpretation, and links back to aims and literature | |
| **Section C: Will the results help locally?** | |
| 1. How valuable is the research? | Yes  No  Can’t Tell |
| *CONSIDER:*   - *If the researcher discusses the contribution the study makes to existing knowledge or understanding (e.g., do they consider the findings in relation to current practice or policy, or relevant research-based literature* - *If they identify new areas where research is necessary* - *If the researchers have discussed whether or how the findings can be transferred to other populations or considered other ways the research may be used*   Findings directly inform service development, identify access barriers, articulate user priorities, and contribute to theory and policy in LMIC prosthetic services. | |

| **APPRAISAL SUMMARY**: *List key points from your critical appraisal that need to be considered when assessing the validity of the results and their usefulness in decision-making.* | | |
| --- | --- | --- |
| **Positive/Methodologically sound** | **Negative/Relatively poor methodology** | **Unknowns** |
| Clear aim and strong justification for qualitative methodology  Co-produced, culturally embedded study design  Recruitment strategy transparent and appropriate to aims  Very detailed description of data collection (settings, procedures, translation, memos)  Reflexivity addressed explicitly (researcher backgrounds, reflexive diary, team discussions)  Rigorous reflexive thematic analysis with multi-analyst verification  Findings richly evidenced with quotations and conceptual clarity  High practical value for designing prosthetic services and technologies in LMICs | Convenience sampling may exclude individuals not attending services (but acknowledged)  Interviews mostly conducted in clinics meaning risk of potential social desirability bias (participants reluctant to criticise services they depend on)  Only one female participant meaning limited gender representation  Heavy representation of transfemoral users may not reflect broader clinic demographics  Family interviews sometimes uneven in depth due to mixed experience and roles | Whether data saturation was formally considered or monitored  Extent to which the presence of researchers or clinic staff influenced participant openness  How findings might differ for non-attenders or rural users not captured in sampling  Long-term impact of co-production on interview dynamics (not fully elaborated) |

**Referencing recommendation:**

CASP recommends using the Harvard style referencing, which is an author/date method. Sources are cited within the body of your assignment by giving the name of the author(s) followed by the date of publication. All other details about the publication are given in the list of references or bibliography at the end.

Example:

*Critical Appraisal Skills Programme (2024). CASP (insert name of checklist i.e. systematic reviews with meta-analysis of randomised controlled trials (RCTs) Checklist.) [online] Available at: insert URL. Accessed: insert date accessed.*

**Creative Commons**

©CASP this work is licensed under the Creative Commons Attribution – Non-Commercial- Share A like. To view a copy of this licence, visit <https://creativecommons.org/licenses/by-nc-sa/4.0/>

**Need further training on evidence-based decision making?** Our online training courses are helpful for healthcare educational researchers and any other learners who:

- Need to critically appraise and stay abreast of the healthcare research literature as part of their clinical duties.
- Are considering carrying out research & developing their own research projects.
- Make decisions in their role, whether that be policy making or patient facing.

**Benefits of CASP Training:**

- Affordable – courses start from as little as £6
- Professional training – leading experts in critical appraisal training
- Self-directed study – complete each course in your own time
- 12 months access – revisit areas you aren’t sure of and revise
- CPD certification - after each completed module

Scan the QR code below or visit <https://casp-uk.net/critical-appraisal-online-training-courses/> for more information and to start learning more.

**
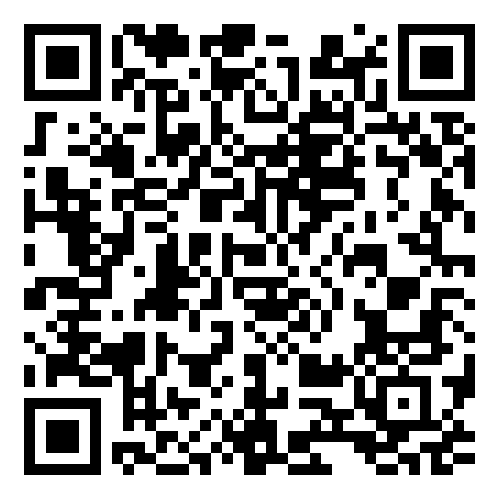
**
