# Supplementary material for: Exploring meaningful outcome domains of recovery following lower limb amputation (LLA) and prosthetic rehabilitation in low- and middle-income (LMIC) settings: a qualitative systematic review
Source: BMJ Open. 2026 Jan 28;16(1):e109817. doi: 10.1136/bmjopen-2025-109817 (PMC12853489; doi:10.1136/bmjopen-2025-109817)
Supplement: online supplemental file 5 [file bmjopen-16-1-s005.docx]

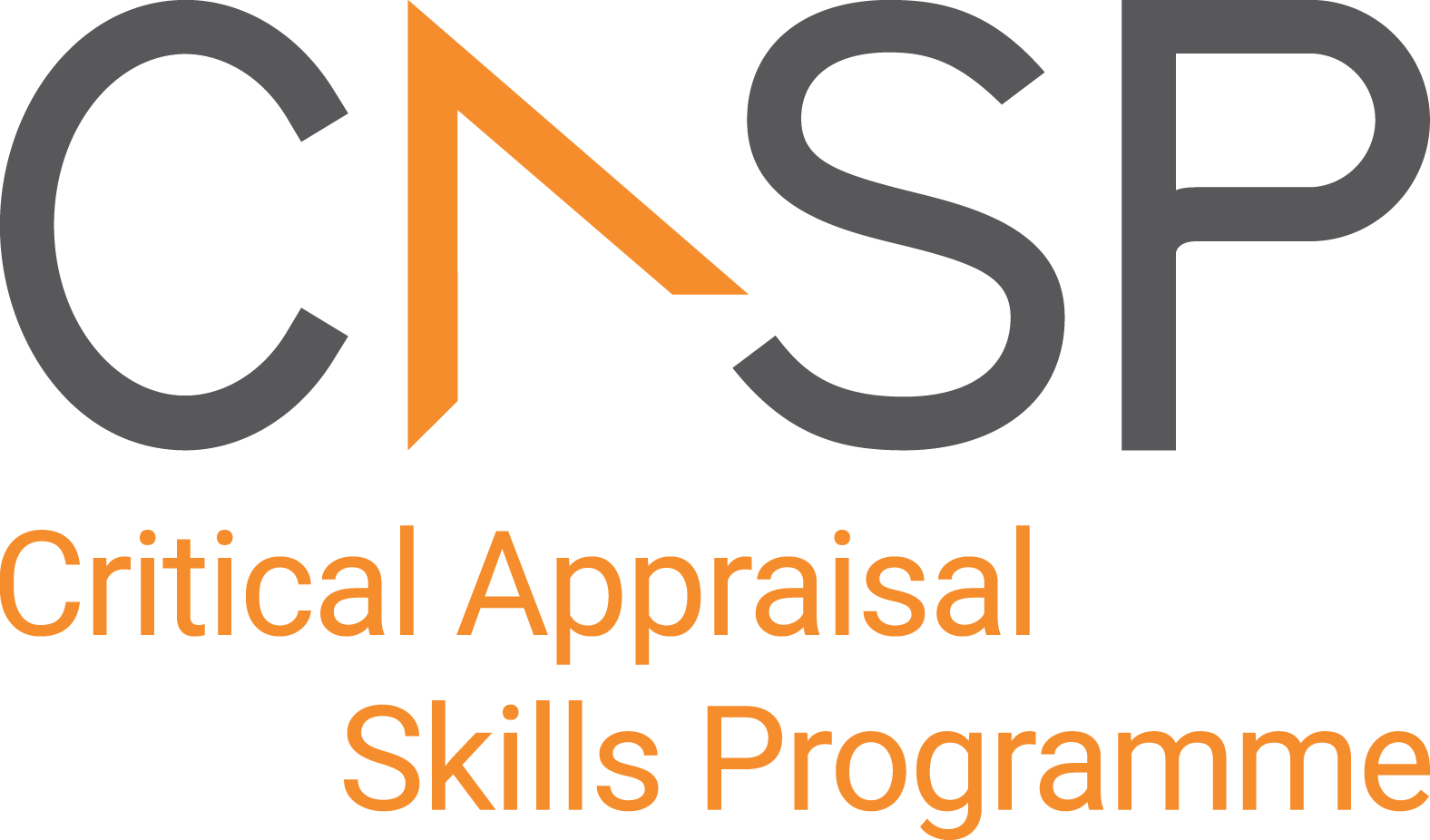
CASP Checklist:

For Qualitative Research

| Paper Title: | Living as a person using a lower-limb prosthesis in Nepal |
| --- | --- |
| **Author:** | Anna Järnhammer, Beatrice Andersson, Prakash Raj Wagle & Lina Magnusson |
| **Web Link:** | https://doi.org/10.1080/09638288.2017.1300331 |
| **Appraisal Date:** | May 2024 |

During critical appraisal, never make assumptions about what the researchers have done. If it is not possible to tell, use the “Can’t tell” response box. If you can’t tell, at best it means the researchers have not been explicit or transparent, but at worst it could mean the researchers have not undertaken a particular task or process. Once you’ve finished the critical appraisal, if there are a large number of “Can’t tell” responses, consider whether the findings of the study are trustworthy and interpret the results with caution.

| **Section A Are the results valid?** | |
| --- | --- |
| 1. Was there a clear statement of the aims of the research? | Yes  No  Can’t Tell |
| *CONSIDER:*   - *what was the goal of the research?* - *why was it thought important?* - *its relevance*   The study clearly states its aim: to explore experiences of people in Nepal using lower-limb prostheses in relation to the Convention on the Right of Persons with Disabilities (CRPD) articles in mobility, education/vocational training, health, rehabilitation and employment | |
| 1. Is a qualitative methodology appropriate? | Yes  No  Can’t Tell |
| *CONSIDER:*   - *If the research seeks to interpret or illuminate the actions and/or subjective experiences of research participants* - *Is qualitative research the right methodology for addressing the research goal?*   The research seeks to explore lived experience and subjective meaning, making qualitative methodology appropriate | |
| 1. Was the research design appropriate to address the aims of the research? | Yes  No  Can’t Tell |
| *CONSIDER:*   - *if the researcher has justified the research design (e.g., have they discussed how they decided which method to use)*   Although semi-structured interviews and content analysis seem broadly suitable, the authors do not explicitly justify why this design was chosen or argue how it best addresses the CRPD-framed aims. Rationale is implied, not articulated | |
| 1. Was the recruitment strategy appropriate to the aims of the research? | Yes  No  Can’t Tell |
| *CONSIDER:*   - *If the researcher has explained how the participants were selected* - *If they explained why the participants they selected were the most appropriate to provide access to the type of knowledge sought by the study* - *If there are any discussions around recruitment (e.g. why some people chose not to take part)*   Participants were selected via convenience sampling from a single rehabilitation centre however the authors do not discuss why this approach was chosen, whether it captured the diversity needed to answer the research question, why non-attenders or non-users were excluded or how recruitment constraints shaped findings. | |
| 1. Was the data collected in a way that addressed the research issue? | Yes  No  Can’t Tell |
| *CONSIDER:*   - *If the setting for the data collection was justified* - *If it is clear how data were collected (e.g. focus group, semi-structured interview etc.)* - *If the researcher has justified the methods chosen* - *If the researcher has made the methods explicit (e.g. for interview method, is there an indication of how interviews are conducted, or did they use a topic guide)* - *If methods were modified during the study. If so, has the researcher explained how and why* - *If the form of data is clear (e.g. tape recordings, video material, notes etc.)* - *If the researcher has discussed saturation of data*   Interviews were structured around CRPD domains, interpreter procedures were described, transcripts were checked, and audio recordings used. | |
| 1. Has the relationship between researcher and participants been adequately considered? | Yes  No  Can’t Tell |
| *CONSIDER:*   - *If the researcher critically examined their own role, potential bias and influence during (a) formulation of the research questions (b) data collection, including sample recruitment and choice of location* - *How the researcher responded to events during the study and whether they considered the implications of any changes in the research design*   There is no discussion of researcher positionality, potential bias, power dynamics, cross-cultural influence, or interpreter effects. Reflexivity is absent. | |
| **Section B: What are the results?** | |
| 1. Have ethical issues been taken into consideration? | Yes  No  Can’t Tell |
| *CONSIDER:*   - *If there are sufficient details of how the research was explained to participants for the reader to assess whether ethical standards were maintained* - *If the researcher has discussed issues raised by the study (e.g. issues around informed consent or confidentiality or how they have handled the effects of the study on the participants during and after the study)* - *If approval has been sought from the ethics committee*   Ethics approvals, consent procedures, confidentiality protocols and reimbursement are clearly described. | |
| 1. Was the data analysis sufficiently rigorous? | Yes  No  Can’t Tell |
| *CONSIDER:*   - *If there is an in-depth description of the analysis process* - *If thematic analysis is used. If so, is it clear how the categories/themes were derived from the data* - *Whether the researcher explains how the data presented were selected from the original sample to demonstrate the analysis process* - *If sufficient data are presented to support the findings* - *To what extent contradictory data are taken into account* - *Whether the researcher critically examined their own role, potential bias and influence during analysis and selection of data for presentation*   The authors describe the steps of content analysis and mention coding and theme development but no detail is given about analyst triangulation, no reflexive examination of coding influence is provided, saturation is not discussed and contradictory cases are not examined. | |
| 1. Is there a clear statement of findings? | Yes  No  Can’t Tell |
| *CONSIDER:*   - *If the findings are explicit* - *If there is adequate discussion of the evidence both for and against the researcher’s arguments* - *If the researcher has discussed the credibility of their findings (e.g. triangulation, respondent validation, more than one analyst)* - *If the findings are discussed in relation to the original research question*   Six themes are clearly presented with strong supporting quotations and linked logically back to the CRPD framework | |
| **Section C: Will the results help locally?** | |
| 1. How valuable is the research? | Yes  No  Can’t Tell |
| *CONSIDER:*   - *If the researcher discusses the contribution the study makes to existing knowledge or understanding (e.g., do they consider the findings in relation to current practice or policy, or relevant research-based literature* - *If they identify new areas where research is necessary* - *If the researchers have discussed whether or how the findings can be transferred to other populations or considered other ways the research may be used*   Despite some limitations in methodological transparency and a lack of explicit policy mapping, the study provides important insight into the lived experience of lower-limb prosthesis users in Nepal, a context where research is extremely scarce. The findings highlight barriers related to mobility, employment, rehabilitation access and stigma, offering a foundational understanding that can inform future research, clinical practice and service development in low-resource settings. Given the dearth of qualitative evidence in LMICs, the study makes a meaningful contribution by documenting user experiences that are otherwise absent from the literature, thereby supporting its value for decision-making and future inquiry. | |

| **APPRAISAL SUMMARY**: *List key points from your critical appraisal that need to be considered when assessing the validity of the results and their usefulness in decision-making.* | | |
| --- | --- | --- |
| **Positive/Methodologically sound** | **Negative/Relatively poor methodology** | **Unknowns** |
| **Clear and explicitly stated research aim aligned with CRPD framework**  **Qualitative methodology appropriate for exploring lived experience**  **Data collection was well described, systematic, and clearly linked to research aims (semi-structured interviews, audio recording, guided by CRPD domains)**  **Ethical processes were robust and transparently reported (dual ethical approval, informed consent, confidentiality measures, travel reimbursements)**  **Findings were clearly presented, well supported by participant quotations, and mapped to CRPD principles**  **Valuable contribution in a context with very limited existing qualitative evidence** | **No reflexivity: researchers did not discuss positionality, influence, interpreter impact, or cross-cultural power dynamics**  **Research design not explicitly justified - authors did not explain why content analysis and interview structure were chosen**  **Recruitment strategy (convenience sampling) not justified or critically examined; excludes non-service users and introduce bias**  **Analytical rigour insufficiently demonstrated: no mention of triangulation, coding verification, saturation, or handling of contradictory cases**  **Single-centre sampling limits transferability** | **True transferability of findings to wider Nepali contexts or LMIC settings**  **How findings might guide service development or rehabilitation planning is not discussed**  **Unclear how interviewer influence or cultural dynamics shaped data**  **Unknown whether saturation was assessed**  **Unknown how coding reliability or analytic checks were conducted** |

**Referencing recommendation:**

CASP recommends using the Harvard style referencing, which is an author/date method. Sources are cited within the body of your assignment by giving the name of the author(s) followed by the date of publication. All other details about the publication are given in the list of references or bibliography at the end.

Example:

*Critical Appraisal Skills Programme (2024). CASP (insert name of checklist i.e. systematic reviews with meta-analysis of randomised controlled trials (RCTs) Checklist.) [online] Available at: insert URL. Accessed: insert date accessed.*

**Creative Commons**

©CASP this work is licensed under the Creative Commons Attribution – Non-Commercial- Share A like. To view a copy of this licence, visit <https://creativecommons.org/licenses/by-nc-sa/4.0/>

**Need further training on evidence-based decision making?** Our online training courses are helpful for healthcare educational researchers and any other learners who:

- Need to critically appraise and stay abreast of the healthcare research literature as part of their clinical duties.
- Are considering carrying out research & developing their own research projects.
- Make decisions in their role, whether that be policy making or patient facing.

**Benefits of CASP Training:**

- Affordable – courses start from as little as £6
- Professional training – leading experts in critical appraisal training
- Self-directed study – complete each course in your own time
- 12 months access – revisit areas you aren’t sure of and revise
- CPD certification - after each completed module

Scan the QR code below or visit <https://casp-uk.net/critical-appraisal-online-training-courses/> for more information and to start learning more.

**
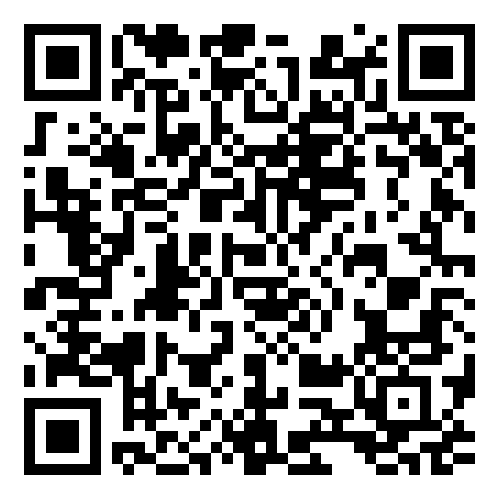
**
