# Supplementary material for: Exploring meaningful outcome domains of recovery following lower limb amputation (LLA) and prosthetic rehabilitation in low- and middle-income (LMIC) settings: a qualitative systematic review
Source: BMJ Open. 2026 Jan 28;16(1):e109817. doi: 10.1136/bmjopen-2025-109817 (PMC12853489; doi:10.1136/bmjopen-2025-109817)
Supplement: online supplemental table 1 [file bmjopen-16-1-s006.docx]

**Supplementary Table 1: Study design and critical appraisal of study quality using the CASP qualitative appraisal tool**  

| **Author** | **Methodology** | **Data Collection approach** | **Analysis approach** | **CASP tool Section A** | | | | | | **CASP tool Section B** | | | **CASP tool Section C** | **Total Score** |
| --- | --- | --- | --- | --- | --- | --- | --- | --- | --- | --- | --- | --- | --- | --- |
|  |  |  |  | **Was there a clear statement of the aims of the research?** | **Is a qualitative methodology appropriate?** | **Was the research design appropriate to address the aims of the research?** | **Was the recruitment strategy appropriate to the aims of the research?** | **Was the data collected in a way that addressed the research issue?** | **Has the relationship between researcher and participants been adequately considered?** | **Have ethical issues been taken into consideration?** | **Was the data analysis sufficiently rigorous?** | **Is there a clear statement of findings?** | **How valuable is the research?** |  |
| Mattick et al. (2023) [23] | Qualitative | SSI | TA |  |  |  |  |  |  |  |  |  |  | **10** |
| Donovan-Hall et al. (preprint) [24] | Qualitative | SSI | TA |  |  |  |  |  |  |  |  |  |  | **11** |
| Lang & Svensk (2018) [25] | Qualitative | SSI | TA |  |  |  |  |  |  |  |  |  |  | **12** |
| Stuckey et al. (2020) [26] | Not stated | SSI | TA |  |  |  |  |  |  |  |  |  |  | **12** |
| Jarnhammer et al. (2018) [22] | Qualitative | SSI | Content |  |  |  |  |  |  |  |  |  |  | **15** |

TA- Thematic analysis,  SSI – Semi structured Interview
