# Supplementary material for: Exploring meaningful outcome domains of recovery following lower limb amputation (LLA) and prosthetic rehabilitation in low- and middle-income (LMIC) settings: a qualitative systematic review
Source: BMJ Open. 2026 Jan 28;16(1):e109817. doi: 10.1136/bmjopen-2025-109817 (PMC12853489; doi:10.1136/bmjopen-2025-109817)
Supplement: Supplementary file 7 [file bmjopen-2025-109817.reviewer_comments.pdf]

PEER REVIEW HISTORY

BMJ Open publishes all reviews undertaken for accepted manuscripts. Reviewers are asked to complete a checklist review form and are provided with free text boxes to elaborate on their assessment. These free text comments are reproduced below.

ARTICLE DETAILS

Title (Provisional)

Exploring meaningful outcome domains of recovery following lower limb amputation and prosthetic rehabilitation in low-and-middle-income (LMIC) settings: A Systematic Review

Authors

Fewins, Caroline Jean; Ostler, Chantel; Donovan-Hall, Margaret

---

VERSION 1 - REVIEW

**Reviewer**	**1**
**Name**	**Sions, Jaclyn**
**Affiliation**	**University of Delaware, Physical Therapy**
**Date**	**06-Sep-2025**
**COI**	**None**

Author are applauded for explicit methodology, enhancing research transparency and replication ability. Strengths include searching reference lists for unidentified articles for inclusion and guideline adherence. Major concerns include:

1. Limited search that did not include PubMed, without acknowledgement of this as a limitation. Given the limited numbers of articles identified, one must question whether a more expansive search would have yielded more results/articles.

2. Seems critical to know how many studies were excluded due to inclusion of both users of prosthesis and individuals who were not using a prosthesis. This remains unclear. Similarly, it is unclear how many studies were excluded that included prosthetic service utilization, which may have had relevant data informing the research question.

3. Due to the interpretation of data, suggest including information about the background of coders to offer insight into their perspectives.

4. Would have liked to see time since amputation as a variable included to characterize the samples. And it remains a bit unclear if the sample is more representative of care-seeking individuals in need of prosthetic care/modifications or individuals living with a prosthesis in these communities, who might be more appropriately considered a sample of convenience

without urgent prosthetic needs. State of need at the time of participation may impact reporting.

5. Figure 1. Would like to see a bit more details regarding why studies were excluded for n=2544 and n=3 boxes and specifically why the 3 reports were not retrieved.

6. Page 16, Lines 42-49: Might consider a bit more discussion about cultural differences that may have influenced pain reporting as well as potential expectations of falling given community environments/infrastructure in the included countries.

7. Use lower limb amputation or LLA throughout for consistency. If not clear that all included individuals had amputations, then perhaps the better term is 'lower limb loss' to be inclusive of individuals with congenital amputations, which may have unique perspectives as compared to those with acquired amputations.

Minor grammatical and clarification suggestions:

1. Abstract: but are limited 'by' five small studies-suggest 'to'

2. Page 3, Line 26: Write out US

3. Page 3, Line 32: recommend 'Xu et al'

4. Page 3, Line 42: unclear what 'role functioning' means exactly. Might provide example(s).

5. Page 3, Line 43: 'for' versus 'in' recommended

6. Page 4, Line 9: LMICs followed by period, not comma

7. Page 5, Line 48: suggest '4' studies rather than 80% to align with the sentence that follows, particularly since the number of studies is <10

8. Page 5, Line 59: what does '2' represent

9. Page 5, Line 59: not sure what is meant by 'due to co-authorship'

10. Conclusion. Seems assumptive to say 'following prosthetic rehabilitation' unless all studies had this as inclusion criteria, as some individuals may have received a prosthesis without 'rehabilitation'.

**Reviewer**	**2**
**Name**	**Hagberg, Kerstin**
**Affiliation**	**University of Gothenburg, Dept of Orthopeadics**
**Date**	**13-Sep-2025**
**COI**	**None**

Congrats to this review and synthesise of qualitative studies focusing on the experience of prosthetic users in low- and middleincome countries, which from my experience is the first

of its kind. I have only a few minor comments on the presentation of the 55 participants (Page 6).

First, I lack some brief information on for how long these 55 individuals had been using a prosthesis at the time for their participation, at least min-max years.

Secondly, from my opinion, presenting decimals of percentages is of no meaning if you can round up or down to a full figure, ie. 73% instead of 72.7% etc. Hope you agree.

**Reviewer**	**3**
**Name**	**Vasanthan, Lenny**
**Affiliation**	**Christian Medical College Vellore**
**Date**	**15-Oct-2025**
**COI**	**None**

Title: “Exploring meaningful outcome domains of recovery following lower limb amputation and prosthetic rehabilitation in low-and-middle-income (LMIC) settings: A Systematic review.”<br>I would suggest the authors to rephrase the title to “Exploring meaningful outcome domains of recovery for persons with lower limb amputation and prosthetic rehabilitation in low-and-middle-income (LMIC) settings: A qualitative systematic review”
Abstract: Well written, there is a typo in Line 26: ‘Tric’ database (also in Table 2)
Background:<br>Line 20, “Without a clear definition of rehabilitation success...” I am not sure of the relevance of this statement here, as successful rehabilitation is determined by individual centres and it varies across the world. Outcomes help us document the services received by patients and for evaluating progress, each rehab centre might have their key performance indicators, even in LMIC.<br>Reference 9, the World Health Organization has launched a recent guideline in 2022, suggest the authors to update this reference.<br>Page 2, Lines 42, 43 says “Identifying patient-defined outcomes in LMICs could better guide resource allocation and enhance value in low-resource prosthetic services” It might be good to add supporting literature for this statement if possible.<br>Lines 50 to 53 states “The review included only studies from the past decade to reflect recent prosthetic advances, but this may have excluded older LMIC research that remains relevant, especially given slower healthcare development and limited access to advanced prosthetics in these settings [11].” I am not sure if this statement is justified enough to perform this review? Unfortunately, the authors could not add any more studies to the existing review (one reference (reference 19) - which was missed in the earlier review, and one unpublished study) (Reference 2). I am not sure how this paper (19) was missed in their earlier review.
Methods:

Reference 12 – is used for both PRISMA and ENTREQ guidelines. May I suggest the authors to please add the accurate reference for PRISMA.<br>Also, add a citation for Rayyan website (page 4, line 24).<br>Line 55,56 mentions reference 15 -17, 18. Can the authors please verify if this is correct usage of these references.<br>There are instances where the authors have used just the percentages, it might be good to also mention the numbers along with the percentages.
Results:<br>Page 8, lines 5,6 mentions two studies, it might be good for the readers to identify which studies are the authors referring to here.<br>Line 10 – the authors have quoted references 15,16. 15 is a landmark article. However, 16 is an unpublished study. Kindly check and verify.<br>In figure 2. The number 1.2 is missing (minor) seems to be a typo here.<br>Table 6 – Please verify the references to the included studies (References 58, 66, 49) looks familiar to the earlier review (2).
Discussion:<br>It might be good to discuss another article that was published earlier, Shashank Ghai, Sander L. Hitzig, Lindsay Eberlin, Joshua Melo, Amanda L. Mayo, Virginie Blanchette, Natalie Habra, Audrey Zucker-Levin, Diana Zidarov, Reporting of Rehabilitation Outcomes in the Traumatic Lower Limb Amputation Literature: A Systematic Review, Archives of Physical Medicine and Rehabilitation, Volume 105, Issue 6, 2024, Pages 1158-1170, https://doi.org/10.1016/j.apmr.2023.08.028.<br>The authors have mentioned about the duration of amputation and gender; it might be worthwhile to explore the treatments received and duration of prosthesis rehabilitation - which also might be crucial to determine outcomes.
Possible typo: Page 18, line 12. Stings and cultures (it possibly might be settings)
Funding: EPSRC TIDAL+ Network (EP/W000717/1, Oldfrey & Dickinson – it might be good to explain what this mean, for the benefit of readers.
Major concerns:<br>No protocol was registered for this review – this is a major concern, as many journals requires the protocol to be published for any systematic review.<br>Two articles (one unpublished article, and one article (possibly missed) in the earlier review) have been identified for this review, not sure how is this justified to have another publication, especially with no prior protocol. (I have highlighted this is to ensure we minimise research waste.)<br>Inclusion of an unpublished study - reference 16. If possible, I suggest the authors to add the paper as preprint and cite the paper.

VERSION 1 - AUTHOR RESPONSE

Location of comment	Reviewer comment	Response
**Reviewer 1**		
Search	Limited search that did not include PubMed, without acknowledgement of this as a limitation.	Thank you for highlighting this.

	Given the limited numbers of articles identified, one must question whether a more expansive search would have yielded more results/articles.	Pubmed was not selected as we chose databases with more complete indexing for qualitative studies. We have added this justification to the text with appropriate references. However, we have also noted this limitation within the Limitations section as suggested.
INC/EXCL	Seems critical to know how many studies were excluded due to inclusion of both users of prosthesis and individuals who were not using a prosthesis. This remains unclear. Similarly, it is unclear how many studies were excluded that included prosthetic service utilization, which may have had relevant data informing the research question.	Thank you for this helpful observation. We have now added specific numbers to the PRISMA description indicating how many full-text studies were excluded because they combined prosthetic users with non-users (without separable qualitative data), and how many were excluded because they examined prosthetic service utilisation only. These exclusions are now explicitly stated in the Results section and reflected in the PRISMA flow diagram.
	Due to the interpretation of data, suggest including information about the background of coders to offer insight into their perspectives.	Thank you for this useful suggestion. We have now added a `Research Team and Reflexivity' section to the Methods outlining our backgrounds and experience Additional information has also been added about the reflexive process undertaken by CF.

	Would have liked to see time since amputation as a variable included to characterize the samples. And it remains a bit unclear if the sample is more representative of care-seeking individuals in need of prosthetic care/modifications or individuals living with a prosthesis in these communities, who might be more appropriately considered a sample of convenience without urgent prosthetic needs. State of need at the time of participation may impact reporting.	We thank the reviewer for this important observation. We reviewed all included studies again and found that time since amputation was inconsistently reported: only one study provided extractable data, while others did not report this variable in a comparable form. We have added this text to the Results section. We have also added a limitation noting that recruitment in all included studies occurred through prosthetic or rehabilitation services, meaning that the samples primarily comprised care-seeking individuals actively engaged with services.
Figure 1	Would like to see a bit more details regarding why studies were excluded for n=2544 and n=3 boxes and specifically why the 3 reports were not retrieved.	Thank you for highlighting this. We have expanded the screening narrative in the Methods section to clarify that title and abstracts were reviewed against the inclusion/exclusion criteria. We also clarify that the three reports listed as 'not retrieved' were unavailable through institutional access, that author contact attempts were unsuccessful, and that no alternative sources (including interlibrary

		routes) yielded the manuscripts. These details are now explicitly reported and align with the PRISMA flow diagram.
Page 16, Lines 42-49:	Might consider a bit more discussion about cultural differences that may have influenced pain reporting as well as potential expectations of falling given community environments/infrastructure in the included countries.	Thank you for this suggestion. We have expanded the Discussion to address how cultural norms around endurance and stoicism may influence participants' reporting of pain and functional difficulties, and how environmental conditions common in many LMICs (such as uneven terrain and limited infrastructure) may shape expectations regarding falls and mobility. We have added supporting references and integrated this material into the Discussion section immediately before the paragraph on the absence of certain outcome domains in the included studies.
Throughout	Use lower limb amputation or LLA throughout for consistency. If not clear that all included individuals had amputations, then perhaps the better term is 'lower limb loss' to be inclusive of individuals with congenital amputations, which may have unique perspectives as compared to those with acquired amputations.	We thank the reviewer for this suggestion. After re-examining all included studies, we confirmed that all participants had acquired lower limb amputations; no congenital limb differences were present. We have therefore standardised

		terminology throughout to “lower limb amputation (LLA)”
Abstract: but are limited 'by' five small studies- suggest 'to'		Abstract has been rewritten as according to Editor’s instructions
Page 3, Line 26	Write out US	Completed with thanks
Page 3, Line 32	recommend 'Xu et al'	Completed with thanks
Page 3, Line 42	unclear what 'role functioning' means exactly. Might provide example(s).	Completed with thanks
Page 3, Line 43	'for' versus 'in' recommended	Completed with thanks
Page 4, Line 9	LMICs followed by period, not comma	Completed with thanks
Page 5, Line 48	suggest '4' studies rather than 80% to align with the sentence that follows, particularly since the number of studies is <10	Completed with thanks
Page 5, Line 59	what does '2' represent	Thank you for highlighting this lack of clarity. We have revised the sentence to specify that the four included studies were independently appraised by two reviewers (CF and CO), with discrepancies resolved through discussion. We also clarified that one study was appraised solely by CF because CO was a co-author of that primary study, and therefore this could introduce a conflict of interest. The revised
Page 5, Line 59	not sure what is meant by 'due to co-authorship'	

		text now makes this explicit.
Conclusion	Seems assumptive to say 'following prosthetic rehabilitation' unless all studies had this as inclusion criteria, as some individuals may have received a prosthesis without 'rehabilitation'.	Thank you for this clarification. We reviewed the included studies and confirmed that while all participants had received a prosthesis, not all had undergone structured prosthetic rehabilitation. We have therefore revised the text to remove the phrase “following prosthetic rehabilitation” and replaced it with “after receiving a prosthesis,” which more accurately reflects the experiences of participants across the included studies.
**Reviewer 2**		
	First, I lack some brief information on for how long these 55 individuals had been using a prosthesis at the time for their participation, at least min-max years.	Thank you for this helpful request. We reviewed all included studies again and found that time since amputation and duration of prosthesis use were reported inconsistently. One study provided extractable data (with a range from a few days to 20 years), while the remaining four did not report this variable in a comparable way. We have added a sentence to the Results section to clarify this and to explain why a min–max range cannot

		be synthesised across all 55 participants.
	Secondly, from my opinion, presenting decimals of percentages is of no meaning if you can round up or down to a full figure, ie. 73% instead of 72.7% etc. Hope you agree.	Thank you for this helpful suggestion. All percentages in the manuscript have now been rounded to whole numbers for clarity and consistency.
**Reviewer 3**		
Background		
Line 20	"Without a clear definition of rehabilitation success..." I am not sure of the relevance of this statement here, as successful rehabilitation is determined by individual centres and it varies across the world. Outcomes help us document the services received by patients and for evaluating progress, each rehab centre might have their key performance indicators, even in LMIC.	Thank you for the perspective. We agree that services often have KPIs (set by service providers/clinicians etc.) which help them to understand and monitor the quality of service they are providing. We argue that this is different to what patients might define as an important outcome of prosthetic provision/rehabilitation and which outcome domains define that success. We were suggesting that if we do not understand what patients think is a successful outcome how can we ensure that our services are providing this and therefore taking a patient centred approach. If we focus on what providers/clinicians think is important we may miss important patient priorities. We have reviewed the text

		and made alteration to ensure this is clearer.
Ref 9	the World Health Organization has launched a recent guideline in 2022, suggest the authors to update this reference.	We have now added the 2022 WHO UNICEF Global Report on Assistive Technology as a reference in the Background, and used its findings to support statements about unmet need and inequities in access to assistive products in LMICs.
Page 2, Lines 42, 43	says "Identifying patient-defined outcomes in LMICs could better guide resource allocation and enhance value in low-resource prosthetic services" It might be good to add supporting literature for this statement if possible.	Thank you for this helpful suggestion. We have now added supporting literature to strengthen this statement. Evidence indicates that incorporating patient-defined priorities improves the relevance of outcome measurement, guides service planning, and supports more efficient resource allocation in LMIC prosthetic and assistive technology contexts [10,11,22,23].
Lines 50 to 53	Lines 50 to 53 states "The review included only studies from the past decade to reflect recent prosthetic advances, but this may have excluded older LMIC research that remains relevant, especially given slower healthcare development and limited<br>access to advanced prosthetics in these settings [11]." I am not sure if this statement is justified enough to perform this review? Unfortunately, the authors could not add any more studies to the existing review (one reference (reference 19) - which was missed in the earlier review, and one unpublished study) (Reference 2). I am not sure	Thank you for this comment. We have reviewed the text and added additional justification for this review, i.e., the concern that including the views of those living in LMICs in a global review risks diluting the lived experiences of individuals in these

	how this paper (19) was missed in their earlier review.	contexts and may overlook social and cultural factors that shape patient priorities for recovery.
Methods		
Reference 12	Reference 12 – is used for both PRISMA and ENTREQ guidelines. May I suggest the authors to please add the accurate reference for PRISMA.	Added in with thanks
Page 4, line 24	Also, add a citation for Rayyan website (page 4, line 24).	Added in with thanks
Line 55-56	Line 55,56 mentions reference 15 -17, 18.<br>Can the authors please verify if this is correct usage of these references.<br><br>There are instances where the authors have used just the percentages, it might be good to also mention the numbers along with the percentages. (i.e. n=2 etc)	We thank the reviewer for this observation. References 16 and 17 were included in error, as these studies do not inform the methodological debate about critical appraisal in qualitative synthesis. We have corrected this by citing the appropriate methodological sources only. The sentence now cites references 14 and 15 for the debate on critical appraisal and 18 specifically for the CASP tool. The manuscript has been updated accordingly. We have also updated the percentages as suggested to numbers for clarity.
Results		
Page 8, lines 5,6	Page 8, lines 5,6 mentions two studies, it might be good for the readers to identify which studies are the authors referring to here.	Thank you for this helpful suggestion. We agree that the referenced studies were not clearly identifiable. We have revised the sentence to

		explicitly name the two studies being referred to improve clarity for the reader.
Line 10	the authors have quoted references 15,16. 15 is a landmark article. However, 16 is an unpublished study. Kindly check and verify	Thank you for highlighting this. Reference 16 was included in error. It is an unpublished empirical study and does not support the methodological point being made. We have removed reference 16 and retained only reference 15 (Braun & Clarke, 2006), which is the appropriate landmark methodological citation. The manuscript has been corrected accordingly
Figure 2	The number 1.2 is missing (minor) seems to be a typo here.	Thank you for drawing our attention to this. The omission of sub-theme number 1.2 was an unintentional typographical error. We have corrected the numbering sequence in the revised Figure 2 so that all sub-themes follow the correct hierarchical structure (1.1–1.4).
Table 6	Please verify the references to the included studies (References 58, 66, 49) looks familiar to the earlier review (2).	Thank you for highlighting this. The reference numbers in the table were carried over in error during drafting and have now been corrected to reflect the numbering in the current review. The updated table accurately cites the

		included LMIC studies. No changes to the manuscript text were required.
Discussion		
	It might be good to discuss another article that was published earlier, Shashank Ghai, Sander L. Hitzig, Lindsay Eberlin, Joshua Melo, Amanda L. Mayo, Virginie Blanchette, Natalie Habra, Audrey Zucker-Levin, Diana Zidarov, Reporting of Rehabilitation Outcomes in the Traumatic Lower Limb Amputation Literature: A Systematic Review,<br>Archives of Physical Medicine and Rehabilitation, Volume 105, Issue 6, 2024, Pages 1158-1170, https://doi.org/10.1016/j.apmr.2023.08.028.<br><br>The authors have mentioned about the duration of amputation and gender; it might be worthwhile to explore the treatments received and duration of prosthesis rehabilitation - which also might be crucial to determine outcomes.	Thank you for this helpful suggestion. We agree that the review by Ghai et al. (2024) is relevant, particularly in highlighting the importance of reporting treatment pathways and the duration of prosthetic rehabilitation when interpreting outcomes. We have now incorporated this reference into the Discussion.<br><br>In the present review, the LMIC studies we included did not consistently report rehabilitation timelines, types of prosthetic interventions received, or duration of prosthesis rehabilitation. These variables therefore could not be synthesised. We now explicitly acknowledge this as an important evidence gap and suggest it as a priority for future LMIC research.
Page 18, line 12	Possible typo: Page 18, line 12. Stings and cultures (it possibly might be settings)	Changed with thanks

	Funding: EPSRC TIDAL+ Network (EP/W000717/1, Oldfrey & Dickinson – it might be good to explain what this mean, for the benefit of readers.	Thank you for this helpful suggestion. We have expanded the funding statement to clarify the nature of the EPSRC TIDAL+ Network, explaining that it is a UKRI-funded research network supporting innovation in assistive technology. This provides clearer context for readers.
Major concerns		
	No protocol was registered for this review – this is a major concern, as many journals requires the protocol to be published for any systematic review.	Thank you raising this important point; please see our earlier comment to the Assistant Editor
	Two articles (one unpublished article, and one article (possibly missed) in the earlier review) have been identified for this review, not sure how is this justified to have another publication, especially with no prior protocol. (I have highlighted this is to ensure we minimise research waste.)	We thank the reviewer for raising this important point. We would like to clarify that this review addresses a fundamentally different research question from the earlier global review undertaken by Ostler et al. (2024). That review synthesised meaningful outcome domains across all settings and was not designed to examine LMIC-specific experiences.<br><br>The present review was undertaken as it was clear findings from the initial review could not be transferred to LMIC setting. We therefore carried out a new, LMIC-specific

		systematic review with a different scope, different inclusion criteria, and a different analytical aim, widening our search and considering an different setting . All studies were independently re-screened against LMIC-specific criteria, and the synthesis focus on this literature enables exploration of contextual, cultural, and infrastructural factors that may have been drowned out in the original review due to the numbers of studies conducted in high income settings.<br><br>Although only two additional LMIC studies were identified, this reflects the extremely limited evidence base in this research area (which is a finding in itself) and demonstrates the need for future empirical research on both the lived experience of prosthetic use in LMICs as well as research exploring outcome domains of importance to people with limb loss. The inclusion of these studies changes the dataset sufficiently to justify an LMIC-focused review and enables an analysis

		that was not possible within the broad, global dataset of the earlier review. We position the work as providing initial insights which could inform future research.
	Inclusion of an unpublished study - reference 16. If possible, I suggest the authors to add the paper as preprint and cite the paper.	Thank you for this helpful suggestion. We agree that citing a preprint is preferable for transparency. This is currently in progress and the reference will be submitted once the preprint has been uploaded to MedRxiv.
